# Influence of Ligands on the Surface Characteristics of CoMo/γ‐Al_2_O_3_ and Hydrodesulfurization Catalytic Activity on Dibenzothiophene‐Type Compounds

**DOI:** 10.1002/open.202400123

**Published:** 2025-03-03

**Authors:** Siphumelele Majodina, Ryan Walmsley, Alisa Govender, Eric C. Hosten, Jaco Olivier, Zenixole Tshentu, Adeniyi S. Ogunlaja

**Affiliations:** ^1^ Department of Chemistry Nelson Mandela University Gqeberha South Africa; ^2^ Research and Development Division Sasol Technology (Pty) Ltd Sasolburg South Africa; ^3^ Materials Division Sasol Technology (Pty) Ltd Sasolburg South Africa; ^4^ Department of Physics and Centre for High Resolution (HRTEM) Nelson Mandela University Gqeberha (Port Elizabeth) South Africa

**Keywords:** Hydrodesulfurization (HDS), Ligands, Molybdenum disulfide (MoS_2_), Dibenzothiophene, CoMo-(L)-γ-Al_2_O_3_ (L=ligands)

## Abstract

Refractory sulfur compounds in fuel oils combust, releasing sulfur oxides (SOx) into the atmosphere, which is a significant source of pollution. In this study, we focused on comparing the surface properties and hydrodesulfurization (HDS) activity of CoMo‐(L)/γ‐Al_2_O_3_ containing chelating ligands (L), specifically acetic acid (AA), with those of ethylenediaminetetraacetic acid (EDTA), citric acid (CA). CoMo/γ‐Al_2_O_3_, CoMo‐AA/γ‐Al_2_O_3_, CoMo‐EDTA/γ‐Al_2_O_3_ and CoMo/γ‐Al_2_O_3_ were prepared by hydrothermal treatment of the mixtures of Co(NO_3_)_2_.6H_2_O and (NH_4_)_6_Mo_7_O_24_.4H_2_O with stoichiometric Co/Mo ratios and enriched with chelating ligands (L=AA, CA and EDTA). Based on the product distributions of the hydrodesulfurization (HDS) of dibenzothiophene (DBT), a reaction pathway of dibenzothiophene (DBT) HDS was proposed to follow hydrogenation (HYD) and direct desulfurization (DDS) routes. In addition, the ligand modification of CoMo/γ‐Al_2_O_3_ catalysts resulted in enhancement of surface properties and HDS activity which is in the order of CoMo‐CA/γ‐Al_2_O_3_ (98 %)> CoMo‐AA/γ‐Al_2_O_3_ (94 %) > CoMo‐EDTA/γ‐Al_2_O_3_ (90 %) > CoMo/γ‐Al_2_O_3_ (43 %). CoMo‐AA/γ‐Al_2_O_3_ presented a higher HYD/DDS ratio compared to CoMo‐CA/γ‐Al_2_O_3_, CoMo‐EDTA/γ‐Al_2_O_3_, and CoMo/γ‐Al_2_O_3_, respectively which makes it a promising HDS catalyst.

## Graphical abstract

study compared CoMo/γ‐Al2O3 catalysts with chelating ligands (acetic acid (AA), ethylenediaminetetraacetic acid (EDTA), citric acid (CA)) for hydrodesulfurization (HDS) of dibenzothiophene. CoMo‐CA/γ‐Al_2_O_3_ showed the highest HDS activity (98%), while CoMo‐AA/γ‐Al_2_O_3_ had the best hydrogenation/desulfurization ratio, making it a promising catalyst.

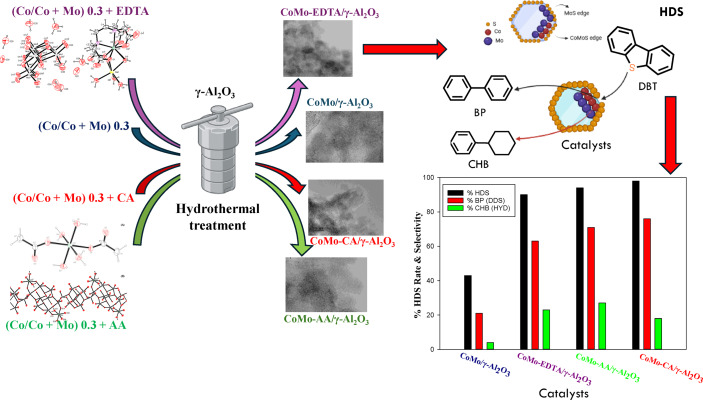

## 1 Introduction

It has been established that the combustion of fossil fuels results in the emission of harmful gases, including sulfur oxides (SOx), into the atmosphere. These emissions can pose risks to both humans and ecosystem and cause significant corrosion in combustion engines.^[1]^ To mitigate the negative impact, stringent environmental regulations mandating fuel sulfur levels of less than 10 ppmS have been implemented to promote ultra‐deep desulfurization for low‐sulfur fuels and to enhance air quality.[^2^, ^3^] Hydrodesulfurization (HDS) is a technique employed by the fuel processing industry to remove refractory sulfur compounds with Co(Ni)Mo/Al_2_O_3_ widely used as catalysts in the HDS processes.^[4]^ Studies have shown that Mo‐based catalysts promoted with Co work best for HDS, while Ni as a promoter favours hydrodenitrogenation (HDN).^[1]^ In addition, H. Topsøe, et al.^[5]^ showed that CoMoS structure was the active structure in the CoMo/γ‐Al_2_O_3_ catalysts while γ‐Al_2_O_3_ as support provides desirable mechanical properties and enhances the interaction and dispersion of active metals such as Mo, Co.[^6^, ^7^, ^8^] Studies have however also shown that CoMo/γ‐Al_2_O_3_ suffer reduced sulfidation which result in less formation of active MoS_2_, which in turn affects the HDS catalytic activity.[^8^, ^9^] A large number of ligands such as citric acid (CA), ethylene diamine tetraacetic acid (EDTA), glycol, nitriloacetic acid (NTA), and Tartaric acid (TA) have been selected and added to HDS catalyst to improve sulfidation efficiency and formation of active phases (Type II CoMoS).[^10^, ^11^, ^12^, ^13^, ^14^, ^15^] Citric acid (CA) and Ethylenediaminetetraacetic acid (EDTA) based CoMo catalysts has been widely reported to provide ultra‐deep HDS for straight‐run gas oil.[^16^, ^17^, ^18^, ^19^, ^20^, ^21^, ^22^, ^23^] Acetic acid (AA) is a relatively inexpensive and readily available chemical. has potential as a chelating agent due to its chemical properties and ability to form complexes with metal ions, there is no literature report of its use in HDS related catalytic reactions. However, its effectiveness in CoMo catalysts for HDS would depend on its ability to influence the sulfidation process and promote the formation of the active Co‐Mo−S phase, similar to other chelating agents.

Therefore, the current study compares the effect of chelating ligands such as EDTA, AA, and CA on the active phase dispersion, morphology, and catalytic activity of CoMo‐based catalysts on HDS reactions of dibenzothiophene (DBT). The catalysts were characterized using BET, PXRD, UV‐Vis, FT‐IR, SEM, EDX, XPS, TEM, and TG‐DSC. Single crystal structures of unsupported CoMo catalysts prepared with acetic acid (CoMo‐AA) and EDTA (CoMo‐EDTA) were determined by single‐crystal X‐ray diffraction.

## Experimental

### Materials

All chemicals used in the study are analytical grade and obtained from Sigma‐Aldrich. These are cobalt(II) nitrate hexahydrate (98 %), ammonium heptamolybdate (99 %), ethylenediaminetetraacetic acid (EDTA), (97 %), citric acid monohydrate (CA), (99.5 %), acetic acid (AA), (99 %), heptane, benzothiophene (98 %), dibenzothiophene (98 %), 4,6‐dimethyldibenzothiophene (97 %), Gamma alumina support (γ‐Al_2_O_3_).

### Synthesis of CoMo Catalysts Prepared with Ethylenediaminetetraacetic Acid (EDTA), Citric Acid (CA), and Acetic Acid (AA)

For the preparation of CoMo/γ‐Al_2_O_3_ (*Unchelated catalyst*), Co(NO_3_)_2_.6H_2_O (3.0 mmol, 0.3536 g) and (NH_4_)_6_Mo_7_O_24_.4H_2_O (1 mmol, 2.4718 g) were added to 30 mL deionized water with (Co/Co + Mo) 0.3 molar ratio. The solution (adjusted to pH 9) was added to the γ‐alumina support (1 g). The resulting mixture was transferred into a Teflon‐lined stainless‐steel autoclave, and then hydrothermally treated at 453 K for 4 h. The product was filtered and washed using deionized water and dried at 393 K for 12 h and calcined at 773 K for 4 h.

With CoMo‐EDTA/γ‐Al_2_O_3_ and CoMo‐CA/γ‐Al_2_O_3_ (*Chelated catalysts*), Co(NO_3_)_2_.6H_2_O (3.0 mmol), (NH_4_)_6_Mo_7_O_24_.4H_2_O (1 mmol), were transferred into 30 mL deionized water (Co/Co+Mo) 0.3 molar ratio, Co : EDTA (1 : 1) molar ratio, and Co : CA (1: 2) molar ratio. The resulting mixture pH was adjusted with ammonia solution to pH=9, and then hydrothermally treated at 453 K for 4 h in the presence of 1 g γ‐Al_2_O_3_.

*CoMo‐AA and CoMo‐EDTA Crystals*: Co(NO_3_)_2_.6H_2_O and (NH_4_)_6_Mo_7_O_24_.4H_2_O were added to 30 mL deionized water with (Co/Co+Mo) 0.3 molar ratio. The solution was adjusted to pH 9 followed by the introduction of chelating ligand Co: CA/EDTA/AA (1 : 2). The resulting mixture was transferred into a Teflon‐lined stainless‐steel autoclave and hydrothermally treated at 453 K for 4 h. A pink solution was obtained, which was then filtered and allowed to evaporate slowly at room temperature, resulting in the formation of pink crystals after 4 to 5 weeks.

### Catalyst Sulfidation and Hydrodesulfurization Measurements

Prior to the catalytic tests, the catalysts sample (2 g) was sulfided using 100 mL heptane solution‐containing 10 wt % of CS_2_ under hydrogen pressure of 4.0 MPa with the temperature of 573 K for 4 h in a Parr reactor. Hydrodesulfurization (HDS) studies of the catalysts were conducted in *n*‐heptane containing 0.22 g of dibenzothiophene (DBT) under a hydrogen pressure of 4.0 MPa at a temperature of 573 K, and maintained for a duration of 6 hours, utilizing a Parr reactor as illustrated in Figure S1. The conversion of DBT was monitored using gas chromatography with flame ionization detection (GC‐FID) and gas chromatography‐mass spectrometry (GC‐MS). The HDS activity of the catalysts was evaluated based on equations provided in the supplementary information (Equations S1 and S2).

### Catalyst Characterization

CoMo‐AA and CoMo‐EDTA crystals were obtained as described in section 2.2 by slowly evaporating the resulting mother liquor for 4–5 weeks to obtain crystals which were studied by Bruker APEX II CCD diffractometer with graphite monochromated MoKα radiation using SHELXL‐2018/3^[24]^ and ORTEP‐3 for windows.^[25]^ The crystal structure diagrams were drawn with ORTEP‐3 for windows.^[25]^ UV‐Vis spectra of catalysts were recorded on a Shimadzu UV vis‐DRS Spectrophotometer UV‐3100 with an MPCF‐3100 sample compartment. The FT‐IR analysis was conducted using a Bruker Tensor 27 platinum ATR‐FTIR spectrometer. Brunauer–Emmett–Teller (BET) Surface area and pore size distribution were measured using Micrometrics TriStar II 3020 Surface Area Analyzer. A Perkin Elmer SCIEX Elan‐6100 ICP‐OES AS‐90 with autosampler was used to determine the concentration of the samples. The Powder X‐ray diffraction (PXRD) patterns were recorded using Bruker D2 powder X‐ray diffractometer using Cu radiation with a Lynxeye detector. All data analysis and Rietveld refinement were done using Topas® V6 software. TGA‐DSC analysis was measured using a Perkin Elmer STA 6000 with a thermocouple sensor Pt−Pt/Rh under nitrogen gas. TESCAN Vegas TS 5136LM was used for EDS and SEM analysis. Sulfided catalysts were analyzed by XPS to identify the chemical species on the surface using Kratos Axis Ultra X‐ray Photoelectron Spectrometer equipped with a monochromatic Al Kα source (1486.6 eV). The base pressure of the system was below 3×10^−7^ Pa. XPS experiments were recorded with a 75 W power source using hybrid‐slot spectral acquisition mode and an angular acceptance angle of +/−20°. Kratos version 2 program was used for XPS data analysis and fitting carried out using a Gaussian line shape. To quantify the relative percentage of Mo^4+^, Mo^5+^, and Mo^6+^ species using XPS in the total Mo component, the following formula was used:

(2.1) $$\left[{Mo}^{4+}\right]\left(\%\right)=\frac{A_{{Mo}^{4+}}}{A_{{Mo}^{4+}}+A_{{Mo}^{5+}}+A_{{Mo}^{6+}}}\times 100\;\%$$

In Equation (2.1), [Mo^4+^] represents the sulfidation degree of the samples, A_Mo_^+^, A_Mo_^5+^, and A_Mo_^6+^ are the areas of the peaks assigned to Mo^4+^, Mo^5+^, and Mo^6+^ species, respectively.^[35]^ The percentage of Co_9_S_8_, CoMoS, and Co^2+^ in the total Co species were calculated by the Equation (2.2:

(2.2) $$\left[CoMoS\right]\left(\%\right)=\frac{A_{CoMoS}}{A_{CoMoS}+A_{{Co}_{9}S_{8}}+A_{{Co}^{2+}}}$$

where [CoMoS] is the sulfidation degree of the samples investigated, and A_CoMoS_, $A_{{Co}_{9}S_{8}}$ , and A_Co_^2+^ are the areas of the peaks assigned to CoMoS, Co_9_S_8_, and Co^2+^ species.^[35]^

TPR was analysed using AutoChem 2920 (Micrometrics, USA). The effluent gas analysis from the reduction experiment was monitored using an MKS Cirrus mass spectrometer. HRTEM was performed using a double‐aberration corrected JEOL JEM‐ARM 200F (Jeol, Italy) operated at 200 kV and equipped with an Oxford Xmax 100 EDS detector and Gatan GIF 965ERS with dual electron‐energy loss spectroscopy (EELS) capability. Scanning transmission electron microscopy in high‐angle annular dark field mode (STEM‐HAADF) was also conducted using JEOL JEM‐ARM 200F. Detailed information is presented in supplementary data sections, see S1 and S2.

## 2 Results and Discussion

### 2.1 Characterization of the Catalysts

The use of chelating ligands in the design of CoMoO_x_ (x=4, 6, etc) catalyst promotes its complexation with cobalt (promoter) which allows molybdenum (active metal) to undergo sulfidation process forming MoS_2_.^[13]^

**Figure 1:**
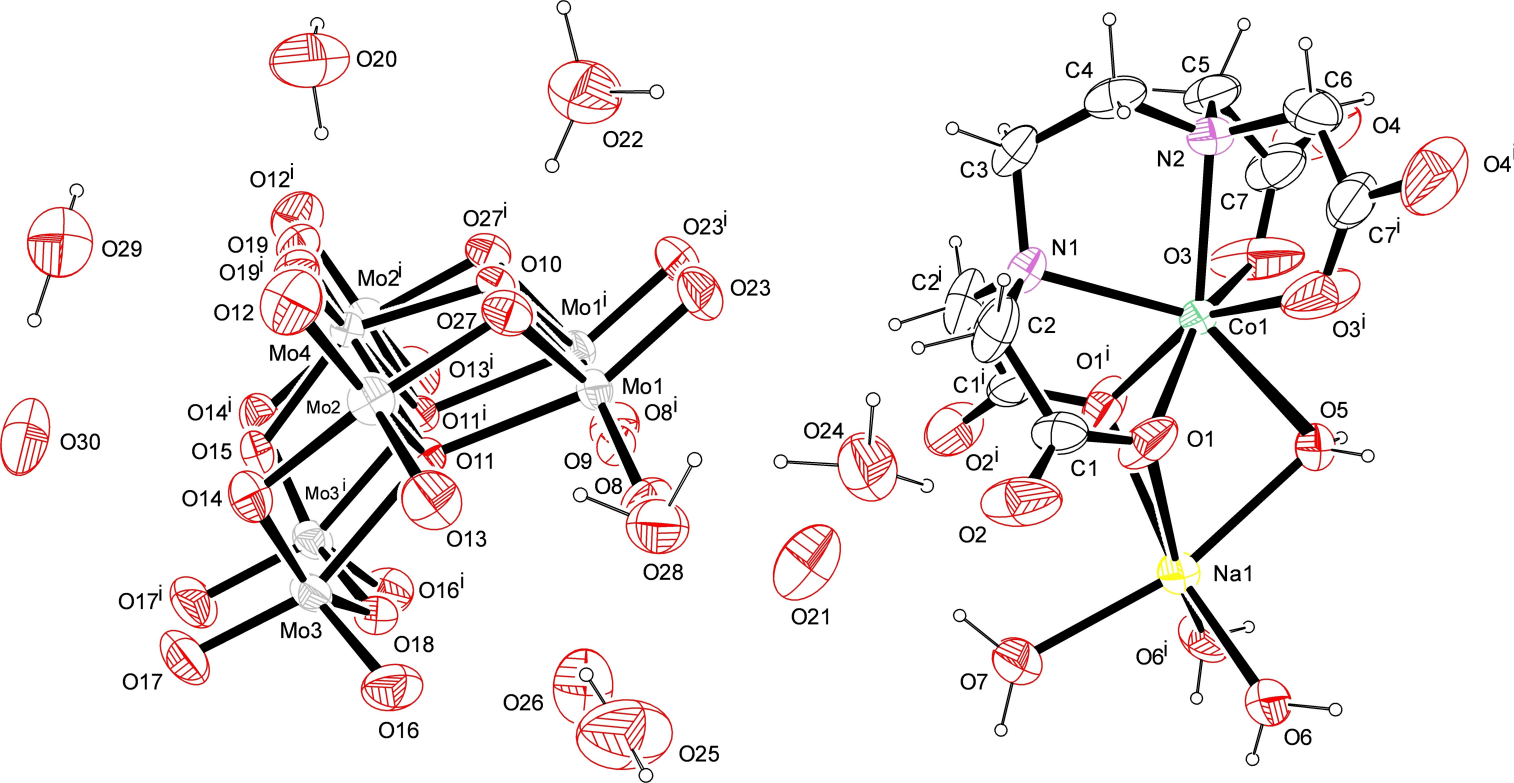
ORTEP diagram of CoMo‐EDTA with ellipsoids drawn at 50 % probability level. Symmetry elements: (i) 1‐x, +y, +z. Selected symmetry generated disorder of the EDTA ligand omitted for clarity.

**Figure 2:**
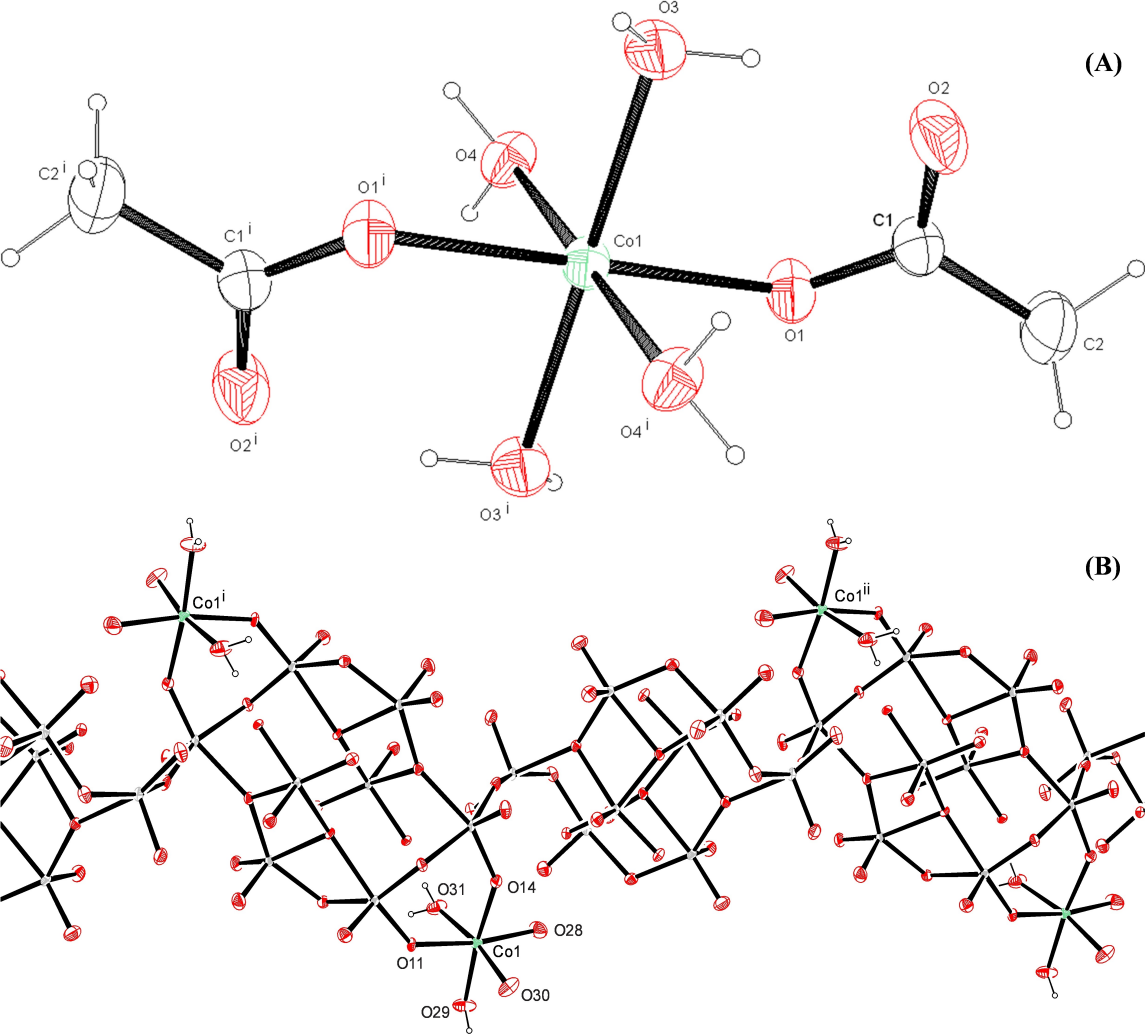
ORTEP diagram of CoMo‐AA showing (A) complexation of Co with AA, and (B) CoO polymeric unit. Ellipsoids were drawn at 50 % probability level. Symmetry elements: (i) 1‐x, 1‐y, 1‐z.

The ORTEP diagram of the crystal structures of CoMo‐EDTA and CoMo‐AA are presented in Figures 1 and 2. CoMo‐EDTA crystallizes in the orthorhombic space group with Pmn21 (No.31). Co atom is coordinated with EDTA in a hepta coordinated–(N_2_O_5_) environment.[^26^, ^27^, ^28^] Bond angle data for Co and Mo as central atoms in CoMo‐EDTA and Hydrogen bond parameters data are presented in Tables S2 and S3, respectively, while CoMo‐AA however, crystallizes in the monoclinic space group with P21/c. The asymmetric unit comprises of one crystallographically independent Co(II) ions complex with acetic acid and water molecules, designated as Co(1) presenting distorted octahedral geometry. The coordination environment of Co(1) was formed by six donors: the equatorial plane constructed by four [O(3), O(3^i^), O(4) and O(4^i^)] (i: 1‐x, 1‐y, 1‐z) donors atoms from water molecules while the axial position were occupied by the remaining two donor atoms originate from acetate ions [O(1) and O(1^i^)], respectively.[^27^, ^28^] Selected bond lengths and angles pertaining to the coordination are tabulated in Tables S4 and S5 and hydrogen bonding are available in Table S6. Data collection parameters and the unit cell are summarized in Table 1. Generally, the formation of Co‐EDTA, further confirms a delay in sulfidation of Co leading to the formation of more MoS_2_ slabs, active phases (CoMoS II).[^13^, ^14^] The formation of cobalt‐ligand complexes (Figure S2) resulted in a delay of Co sulfidation, thus, promoting the formation of active phase (Type II CoMoS) catalyst. CoMo‐AA and CoMo‐EDTA were observed to form rod‐like and rhombic shaped structures, respectively (Figure S2). A detailed interpretation of single crystal data of CoMo‐EDTA and CoMo‐AA are provided in the supplementary data section (section S3).

**Table 1:** Crystal data and details of CoMo‐EDTA and CoMo‐AA.

Compound	CoMo‐EDTA	CoMo‐AA(A)	CoMo‐AA(B)
Empirical formula	Mo_7_O_24_,C_10_H_20_CoN_2_NaO_12_,12(H_2_O), 3(O)	C_4_H_14_CoO_8_	2 (Co H_4_ Mo_8_ O_31_), 2 (C_050_ O_0. 50_), 4 (CO), 14 (O), 4(H_2_ O)
Formula weight	1761.97	296	3089.08
Temperature (K)	296	296	200
Wavelength (Å)	0.71073	0.71073	0.71073
Crystal system	Orthorhombic	Monoclinic	Triclinic
Space group	Pmn21 (No.31)	P21/c	P‐1 (No.2)
D (cal) [g/cm3]	2.300	1.715	2.850
Volume (Å3)	2544.0(3)	482.29(3)	1800.06(13)
Z	2	2	1
a (Å)	16.3617(13)	4.8029(2)	
b (Å)	9.6950(7)	11.9147(5)	10.2722(4)
c (Å)	16.0376(12)	8.4514(3)	13.9640(6)
α (°), β (°), ɣ (°)	90, 90, 90	90, 94.274(2), 90	14.2545(6)
F(000)	1716	258	62.870(2), 87.074(2), 81.630(2)
Crystal size (mm)	0.32×0.39×0.55	0.21×0.30×0.37	1452
θ range for data collection (°)	1.8, 28.3	3.0, 28.3	0.05×0.22×0.23
Dataset h/k/l	−21,21/−12,12/−21,21	−6,6/−15,15/−10,10	1.6, 28.4
Tot., Uniq Data, R (int)	49019, 6542, 0.021	8923, 1200, 0.024	−13,13/−16,18/0,19
Observed Data [I >2.0 σ(I)]	6265	1080	8964, 8964, 0.00
			7947
Nref, Npar	6542, 369	1200, 79	
Final indices [R, wR2, S]	0.0179, 0.0474, 1.10	0.0178, 0.0496, 1.05	8964, 499
Largest peak and hole (eÅ‐3)	−0.48, 0.62	−0.21, 0.25	0.0334, 0.0841, 1.06
			−0.98, 1.54

**Figure 3:**
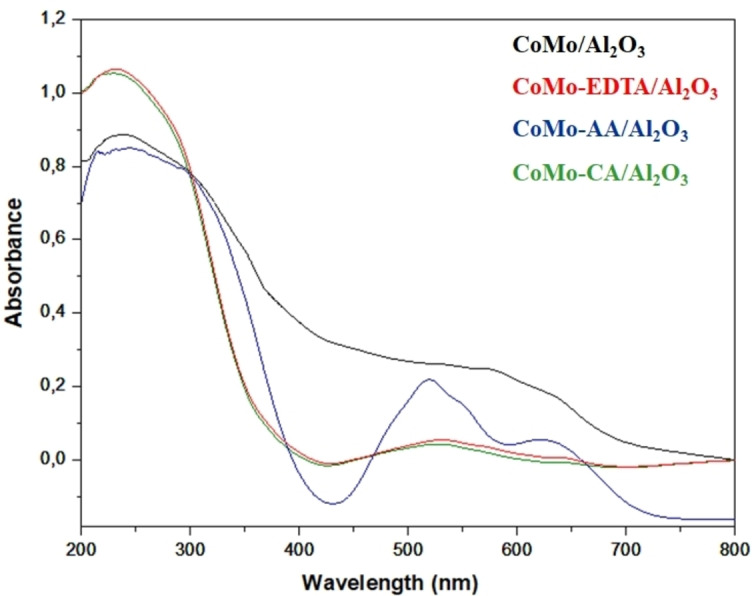
UV‐Vis spectra for CoMo/γ‐Al_2_O_3_, CoMo‐x/γ‐Al_2_O_3_ (x=EDTA, AA, CA) catalysts.

The UV‐Vis absorption spectra show various coordination environments of Co^2+^ and Mo^6+^ ions in CoMo/γ‐Al_2_O_3_, CoMo‐EDTA/γ‐Al_2_O_3_, CoMo‐AA/γ‐Al_2_O_3_, and CoMo‐CA/γ‐Al_2_O_3_ presented in Figure 3, respectively. All catalysts exhibited absorption band at 200–350 nm which are associated with ligand metal‐charge transfer (LMCT) involving O^2−^→Mo^6+^ ions having octahedral coordination in polymeric Mo species.[^10^, ^43^, ^44^, ^45^] The first absorption band for CoMo/γ‐Al_2_O_3_ was observed at 340 nm, whereas the bands for CoMo‐EDTA/γ‐Al_2_O_3_, CoMo‐AA/γ‐Al_2_O_3_, and CoMo‐CA/γ‐Al_2_O_3_ were detected within the range of 231–239 nm.^[44]^ The shift of Mo absorption edge absorption band to a lower wavelength suggests the formation of less polymerized molybdates^[10]^ which could lead to a decrease in catalyst particle size and agglomeration of Mo species.[^10^, ^19^, ^26^, ^36^, ^37^, ^38^, ^39^, ^40^, ^42^, ^46^, ^47^] The absorption band at 500–750 nm region is associated with a ligand field transition of Co(II) ions in both α and β‐CoMoO_4_ compounds,^[29]^ and the Co species assigned to CoAl_2_O_4_ in T_d_ coordination.[^10^, ^30^] The decrease in band intensities of CoMo‐EDTA/γ‐Al_2_O_3_ and CoMo‐CA/γ‐Al_2_O_3_ at 545, 585, and 630 nm compared to CoMo‐AA/γ‐Al_2_O_3_ suggests an increase in cobalt coordination within an octahedral environment.^[31]^ Additionally, a broad band observed around (708–750 nm) has been assigned to Co_3_O_4_.^[36]^

The E_g_ for CoMo/γ‐Al_2_O_3_, CoMo‐EDTA/γ‐Al_2_O_3_, CoMo‐AA/γ‐Al_2_O_3_, and CoMo‐CA/γ‐Al_2_O_3_ are ~3.2 eV, ~3.9 eV, ~3.5 eV, and ~3.9 eV as shown in (Figure S3). The higher E_g_ implies a gradual decrease of the average particle size of Mo species present in the catalyst.[^38^, ^47^, ^48^] The N_Mo_ for CoMo/γ‐Al_2_O_3_, CoMo‐EDTA/γ‐Al_2_O_3_, CoMo‐AA/γ‐Al_2_O_3_, and CoMo‐CA/γ‐Al_2_O_3_ are ~3.9, ~2.6, ~1.3, and ~1.3, respectively (Table 2). The number of neighbouring Mo centers (N_Mo_) when calculated using the formula (N_Mo_=16–3.8E_g_)[^38^, ^47^] shows a linear inverse correlation between the E_g_ value and the number of bridging Mo−O−Mo covalent bonds (calculated using (_NMo‐O‐Mo_=11.8–26E_g_) around the central Mo^6+^ cation (N_Mo‐O‐Mo_), which determines the molecular structures of MoO_x_/Mo_y_O_x_ species were present[^22^, ^47^] (Table 2).

**Table 2:** Band gap energy and the average number of bridging Mo‐O−Mo covalent bonds around the central Mo^6+^ cation (N_Mo‐O‐Mo_) as estimated from the UV‐Vis E_g_ for the oxide CoMo catalysts (N_Mo‐O‐Mo_=(11.8‐2.6E_g_)).

Catalyst	E_g_ values	N_Mo‐O‐Mo_	Structural assignments	Ref.
CoMo/γ‐Al_2_O_3_	3.2	3.5	MoO_3_, [Mo_7_O_24_]^6−^, [Mo_8_O_26_]^4−^	[^22^, ^47^]
CoMo‐AA/γ‐Al_2_O_3_	3.5	2.6	[MoO_4_]^2−^/[Mo_7_O_24_]^6−^, [Mo_7_O_24_]^6−^, [MoO_4_]^−2^	[^22^, ^47^]
CoMo‐EDTA/γ‐Al_2_O_3_	3.9	1.8	[MoO_4_]^2−^/[Mo_7_O_24_]^6−^, MoO_4_/MoO_6_, Mo_2_O_7_	[^22^, ^47^]
CoMo‐CA/γ‐Al_2_O_3_	3.9	1.7	[MoO_4_]^2−^/[Mo_7_O_24_]^6−^, MoO_4_/MoO_6_, Mo_2_O_7_	[^22^, ^47^]

The FT‐IR spectra of synthesized CoMo/γ‐Al_2_O_3_, CoMo‐AA/γ‐Al_2_O_3_, CoMo‐EDTA/γ‐Al_2_O_3_ and CoMo‐CA/γ‐Al_2_O_3_ are presented in Figure S4. The band at 798 and 753 cm^−1^ was attributed to the Mo−O−Mo vibration. The Mo=O stretching vibration is located at 932 cm^−1^.^[49]^ The band at 1588 cm^−1^ in chelated material corresponds to –COO^−^ stretch preserved after impregnation,[^50^, ^51^] and those at 1403.7 cm^−1^ was attributed to hydrogen bonded −COO^−^ vibrations due to the presence of water. The band present at 1385 cm^−1^ is associated with the interaction between –COO^−^ and ‐OH groups on the support[^32^, ^52^] The vibration band at 978 cm^−1^ was assigned to the Mo−N band of CoMo‐EDTA/γ‐Al_2_O_3_. The band found in the 430–450 cm^−1^ and 510 cm^−1^ regions are assigned to the O−Mo−O terminal stretching.[^33^, ^41^, ^42^] The vibration band at 786 cm^−1^ was associated with Mo−O−Mo bridge stretching and can also be assigned to the vibrations of Co−O_6_ band and water liberations.^[43]^ The band at 521, 612, 628, and 622 cm^−1^ are attributed to Mo−O−Mo stretching. The IR confirms the chelating effect of EDTA, AA, and CA on CoMo. The FTIR of sulfided catalysts (**Figure** **S5**), show a disappearance of −COO^−^ vibration bands upon sulfidation.

The surface area and pore volume distribution sulfided CoMo based catalysts are reported in Table 3. N_2_ Adsorption‐desorption isotherms of chelated and non‐chelated CoMo/γ‐Al_2_O_3_ catalysts are given in Figure S6. CoMo‐AA/γ‐Al_2_O_3_ presented pore size distribution pattern with impacts the accessibility of DBT to the active sites within the catalyst (Figure S6B). The hysteresis loops are in the range of relative pressure (P/Po)=0.6–0.9 Å and are type H_2_,^[53]^ indicating the presence of a mesoporous structure type. CoMo/γ‐Al_2_O_3_ gave surface area and average pore size of 17 m^2^/g and 34 Å, respectively, while slightly higher surface areas with corresponding pore volumes was observed for CoMo‐AA/γ‐Al_2_O_3_, CoMo‐EDTA/γ‐Al_2_O_3_ and CoMo‐CA/γ‐Al_2_O_3_ (Table 3). The addition of ligands (AA, CA, EDTA) to CoMo/γ‐Al_2_O_3_ led to improved molecular interaction resulting in enhanced metal dispersion on the support.[^53^, ^54^, ^55^, ^56^, ^57^, ^58^, ^59^, ^60^]

**Table 3:** Textural properties of Al_2_O_3_ and CoMo catalysts.

Sample	BET surface area	Average pore size	ICP‐OES	
	(m^2^ g^−1^)	(Å)	Co Metal (wt.%)	Mo Metal (wt.%)
CoMo/γ‐Al_2_O_3_	17	34	1.13	7.49
CoMo‐EDTA/γ‐Al_2_O_3_	23	41	1.38	7.34
CoMo‐AA/γ‐Al_2_O_3_	20	44	1.25	7.45
CoMo‐CA/γ‐Al_2_O_3_	24	45	1.00	7.38

**Figure 4:**
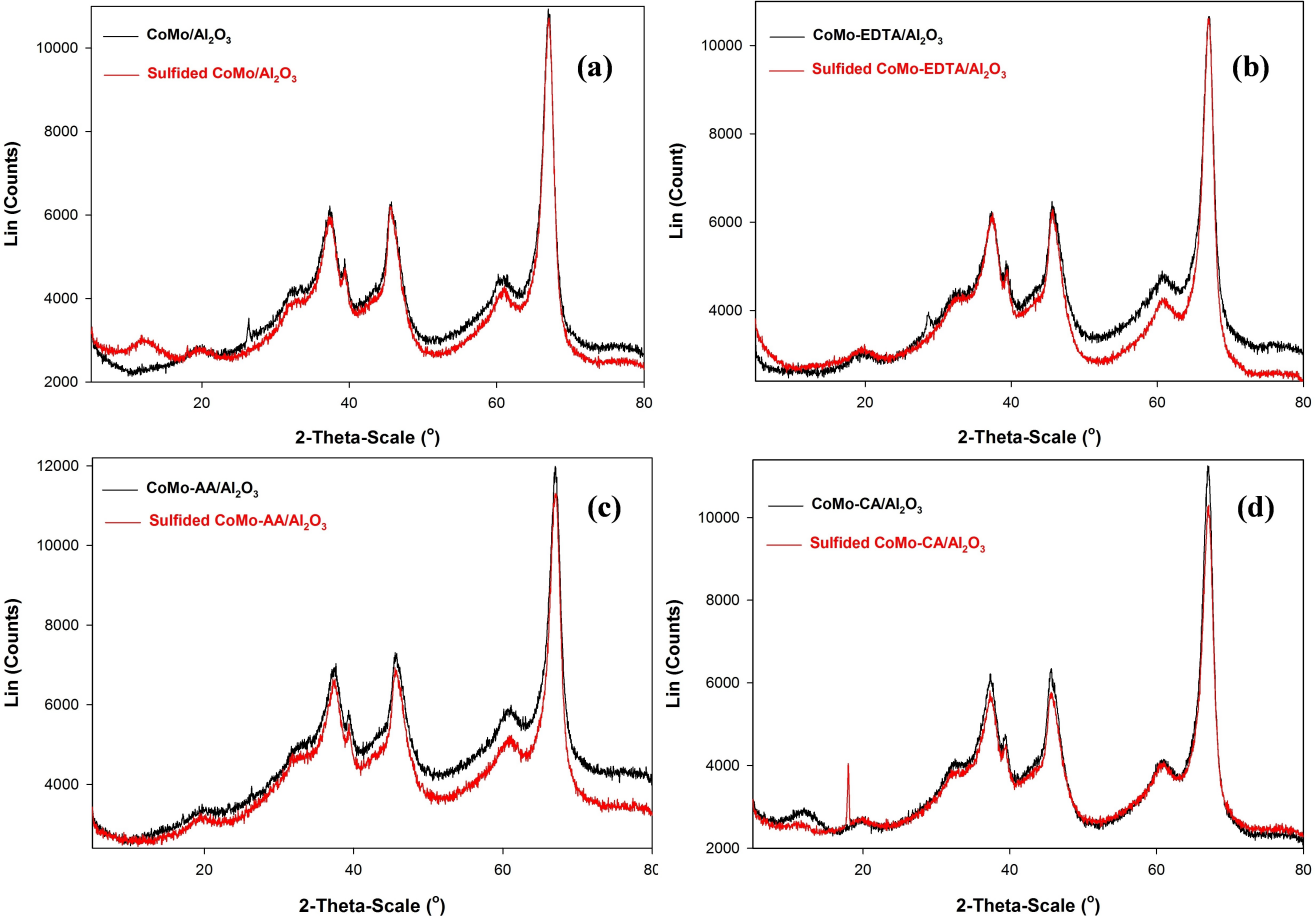
XRD diffraction pattern of oxide and sulfided (a) CoMo/γ‐Al_2_O_3_, (b) CoMo‐EDTA/γ‐Al_2_O_3_, (c) CoMo‐AA/γ‐Al_2_O_3_, and (d) CoMo‐CA/γ‐Al_2_O_3_ catalysts.

The PXRD patterns of CoMo/γ‐Al_2_O_3_ and CoMo‐x/γ‐Al_2_O_3_ (x=EDTA, AA, and CA) for oxide and sulfided catalysts are shown in Figure 4. The diffraction patterns of all the catalysts exhibited similarities, characterized by relatively broad peaks throughout the analysis range, akin to those documented in the literature for the γ‐Al_2_O_3_ defect structure.[^61^, ^62^, ^63^, ^65^] Small sharp reflections at 2θ=26.4° in CoMo/γ‐Al_2_O_3_ and CoMo‐AA/Al_2_O_3_ corresponds to (0 0 2) crystalline monoclinic β‐CoMoO_4_ phase. CoMo‐EDTA/γ‐Al_2_O_3_ exhibited β‐MoO_3_ characteristic peak at 28.7°,^[62]^ while broad diffraction pattern at 12.8° for CoMo‐CA/γ‐Al_2_O_3_ corresponds to β‐MoO_3_ phase. The XRD patterns of sulfided CoMo catalysts (chelated) presented similar reflections confirming a higher level of sulfidation, while sulfided CoMo/γ‐Al_2_O_3_ suggest partial sulfidation due to the presence of broad diffraction peak at 2θ=12.8°.

XPS analysis was performed to investigate the chemical species^[54]^ present on the surface of the sulfided CoMo/γ‐Al_2_O_3_, CoMo‐EDTA/γ‐Al_2_O_3_, CoMo‐AA/γ‐Al_2_O_3_, and CoMo‐CA/γ‐Al_2_O_3_ catalysts. The survey scan spectrum shown in Figure S7(a–d) demonstrated the presence of the key elements, S 2p, Co 2p, Mo 3 d, O 1s, C 1s, Al 1p, Al 2 s elements, respectively. The binding energies caused by peak splitting due to different oxidation states are between 775–805 eV and 224–238 eV are ascribed to Co 2p and Mo 3d spectra, respectively (Table 4).[^59^, ^63^, ^64^] Binding energies of various elements in CoMo/γ‐Al_2_O_3_, CoMo‐EDTA/γ‐Al_2_O_3_, CoMo‐AA/γ‐Al_2_O_3_, and CoMo‐CA/γ‐Al_2_O_3_ are shown in Table 4.

**Table 4:** Binding energies determined in XPS experiments for CoMo/γ‐Al_2_O_3_ and CoMo‐EDTA/γ‐Al_2_O_3_.

Elements (eV)	CoMo/γ‐Al_2_O_3_	CoMo‐EDTA/γ‐Al_2_O_3_	CoMo‐AA/γ‐Al_2_O_3_	CoMo‐CA/γ‐Al_2_O_3_
C 1s	282.2	282.5	282.7; 286.4	283.1
O 1s	529.5	529.0	529.8	529.9
Mo 3d	230.2; 233.5; 226.7	226.0;230.1; 233.4	230.2; 226.4	233.8; 230.6; 227.0
Co 2p	780.5; 795.0	778.9; 793.1	776.9; 778.9; 791.5; 794.0	777.2; 783.5; 780.0
S 2p	160.8; 166.7	159.5; 166.8	162.0; 167.5	161.0; 167.7
Al 2p	72.2	72.1	–	–
Al 2s	117.1	116.4	117.8	117.8

**Figure 5:**
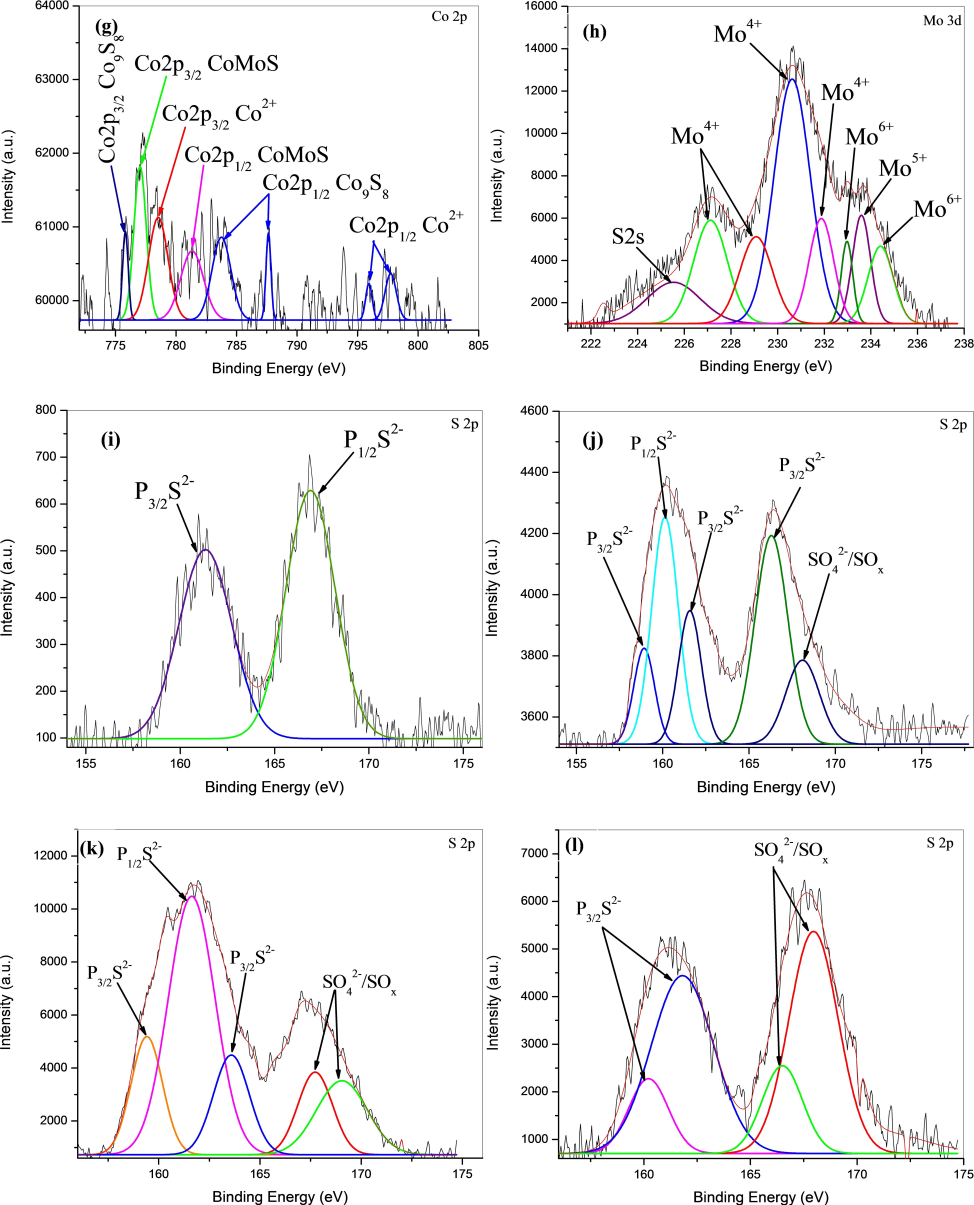
XPS deconvolution of CoMo/γ‐Al_2_O_3_, CoMo‐EDTA/γ‐Al_2_O_3_, CoMo‐AA/γ‐Al_2_O_3_,and CoMo‐CA/γ‐Al_2_O_3_ catalysts, where (a) Co 2p, (b) Mo 3 d, and (i) S 2p is for CoMo/γ‐Al_2_O_3_, (c) Co 2p Mo, (d) 3d, and (j) S 2p is for CoMo‐EDTA/γ‐Al_2_O_3_, (e) Co 2p, (f) Mo 3d, and (k) S 2p is for CoMo‐AA/γ‐Al_2_O_3_, (g) Co 2p, (h) Mo 3d, and (l) S 2p is for CoMo‐CA/γ‐Al_2_O_3_.

The various oxidation state measurements for CoMo/γ‐Al_2_O_3_, CoMo‐EDTA/γ‐Al_2_O_3_, CoMo‐AA/γ‐Al_2_O_3_, and CoMo‐CA/γ‐Al_2_O_3_ were carried out using Origin software to quantify and evaluate Mo 3d as MoS_2_ (Mo^4+^ 3d_5/2_, 3d_3/2_), MoO_x_S_y_ (Mo^5+^ 3d_5/2_, 3d_3/2_), MoO_x_ (Mo^6+^ 3d_5/2_, 3d_3/2_), and sulfide (S^2−^).^[54]^ Co 2p core‐level spectra are also decomposed into three contributions, corresponding to Co 2p_3/2_ and Co 3p_1/2_, evaluated as Co_9_S_8_, CoMoS, and Co^2+^ in oxidic environment (CoO_x_ and CoAl_2_O_4_) phases, respectively.[^39^, ^40^] For Mo^4+^ sulfide (3d_5/2_ BE=228.8–229.1 eV; 3d_3/2_ BE=232.0–232.1 eV), Mo^5+^ oxysulfide (3_d/5_ BE=229.6–230.5 eV; 3d_2/3_ BE =232.9–233.4 eV), and Mo^6+^ oxide (3d_5/2_ BE=232.0–233.0 eV; 3d_3/2_ BE=235.3–236.0 eV).[^67^, ^68^, ^69^, ^70^, ^71^, ^72^] From literature, Co 2p XPS of CoO_x_ (Co^2+^) occurs between 780.8–793.1 eV, Co_9_S_8_ between 777.6–793.2 eV, and CoMoS between 778.3–793.9 eV, while the contribution of S 2 s to Mo 3d is in the BE range of 225.9–226.3 eV. as shown in Figure 5 
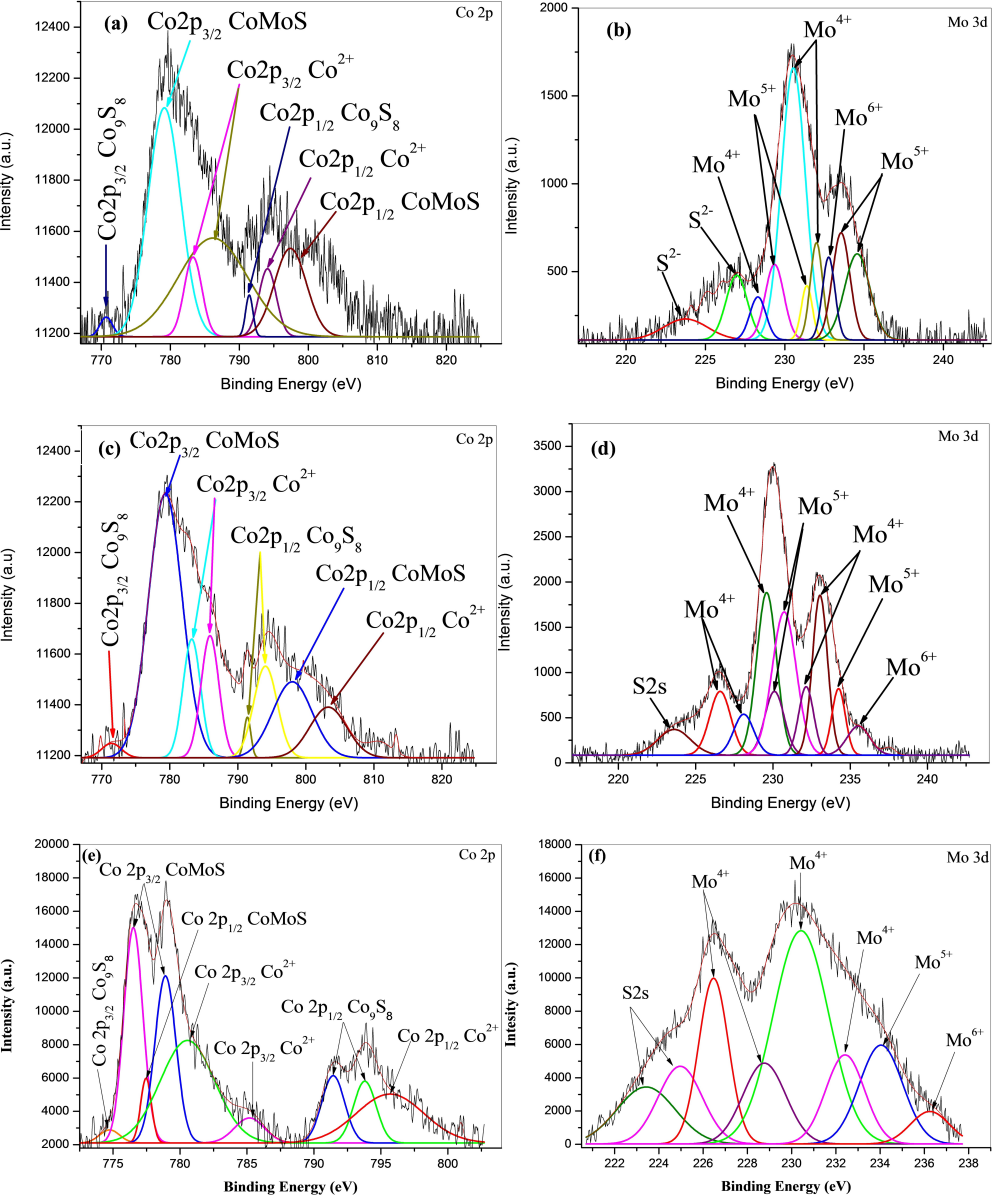
 5a through Figure 5f, also fall within the region.[^16^, ^70^, ^74^]

**Figure 6:**
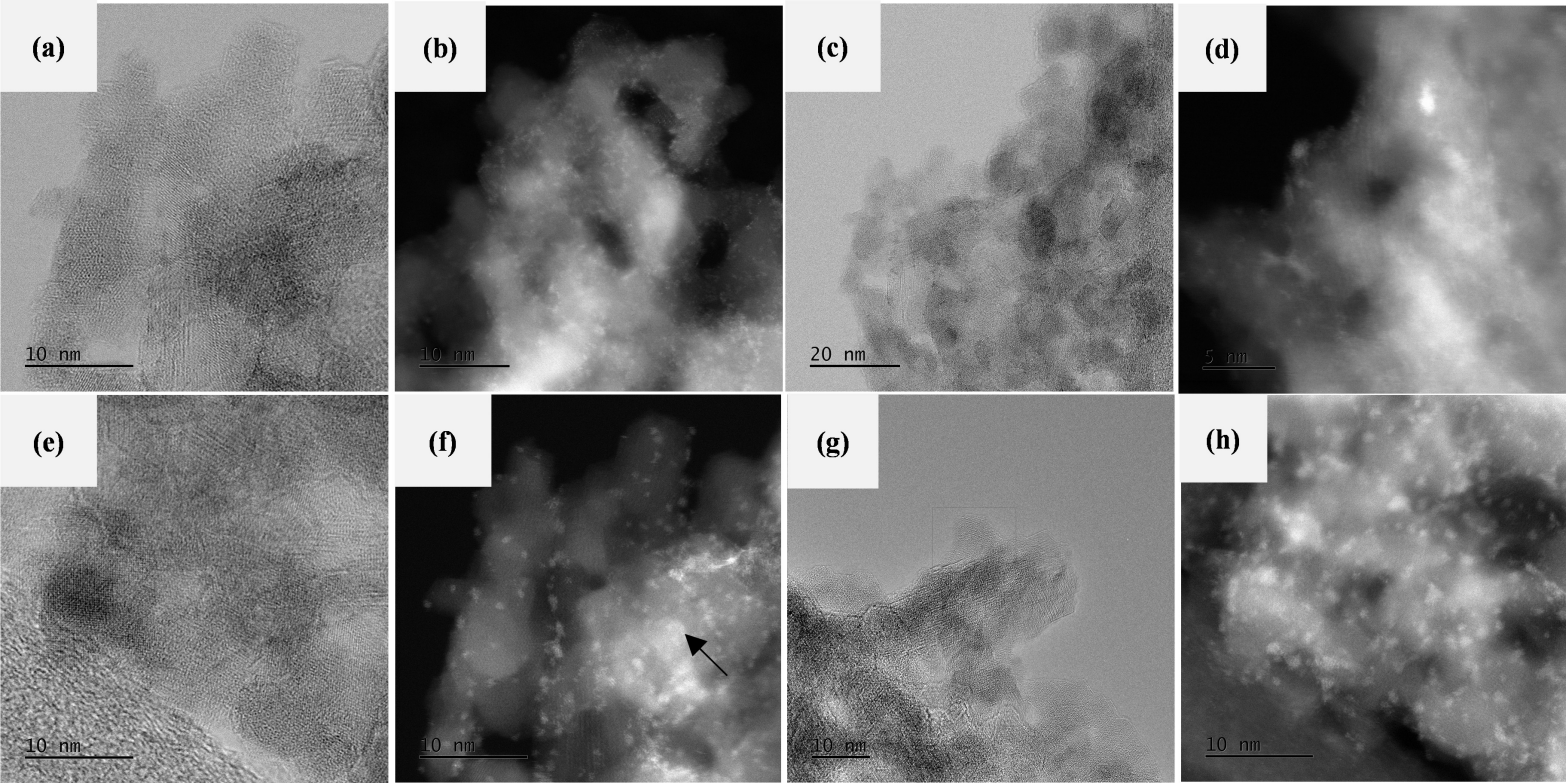
HRTEM images for sulfided (a) CoMo/γ‐Al_2_O_3_; (c) CoMo‐EDTA/γ‐Al_2_O_3_; (e) CoMo‐AA/γ‐Al_2_O_3_ and (h) CoMo‐CA/γ‐Al_2_O_3_. Figures (b), (d), (f) and (h) are STEM‐HAADF images of the corresponding Co and Mo distribution marked in the HRTEM image (bright dotted areas) for CoMo/γ‐Al_2_O_3_; CoMo‐EDTA/γ‐Al_2_O_3_; CoMo‐AA/γ‐Al_2_O_3_ and CoMo‐CA/γ‐Al_2_O_3_, respectively.

Two doublets corresponding to the S 2p_3/2_, S 2p_1/2_, terminal S_2_^2−^ from oxysulfide, and SO_4_^2−^ are observed. S 2p_3/2_ peak at 162.5 eV can be assigned to S^2−^, while S 2p_1/2_ with the binding energy of 162.1 eV can be attributed to polysulfide (S^2−^) (Figure 5e). The S 2p _3/2_ and S 2p_1/2_ for CoMo‐EDTA/γ‐Al_2_O_3_ showed characteristic peaks at 162.1 eV, 162.7 eV, and the characteristic peaks of SO_4_^2−^ were also observed at 163.2 and 167.1 eV (Figure 5f).^[73]^ CoMo‐AA/γ‐Al_2_O_3_ show two characteristic peaks attributed to S 2p_3/2_ as S^2−^, S 2p_1/2_ as S^2−^, and SO_4_^2−^ with binding energies of 162.0 eV and 176.5 eV, respectively. While CoMo‐CA/γ‐Al_2_O_3_ showed characteristic peaks at 161.0 eV and 167.7 eV, attributed to S p_3/2_ (S^2−^) and SO_4_^2−^. All the deconvoluted Mo 3d spectra include the S 2 s peaks at 225.5, 226.0, and 226.1 eV, respectively.^[74]^ The addition of chelating ligand resulted in a shift of Mo 3d_5/2_ and Mo 3d_3/2_ (Table 5) and Co 2p_3/2_ and Co 2p_1/2_ (Table 6). CoMo/γ‐Al_2_O_3_ resulted in lower percentage of Mo^4+^ and CoMoS, while the percentage of Mo^4+^ increases for the chelated catalysts^[34]^ (Table 7). The decrease in binding energy for the chelated catalysts reflects that the electron‐rich Mo species has formed, and it also gives direct evidence for the transfer of electrons from the conductive band to the active Mo. This electron transfer effect weakened the Mo−S bond and promoted the generation of CUS, resulting in enhanced HDS activity^[100]^ as seen in Figure S12. As shown in Figure 6, high concentration of Co observed for CoMo‐AA/γ‐Al_2_O_3_ (Co=1.25 wt %) induced the formation of more Co_9_S_8_ (Figure 6e) over the CoMoS phase. The observed shift in XPS BEs data of MoSx (0 0 2) in CoMo/γ‐Al_2_O_3_ and CoMo−L/γ‐Al_2_O_3_ (L=AA, CA, EDTA) confirmed changes in environment due to the incorporation of ligands.[^76^, ^77^, ^78^, ^79^]

**Table 5:** XPS parameters of the different distributions (BE) of Mo 3 d obtained for unchelated and chelated CoMo/γ‐Al_2_O_3_ catalysts.

Catalysts	BE (eV) Mo 3d_5/2_			BE (eV) Mo 3d_3/2_		
	Mo^4+^	Mo^5+^	Mo^6+^	Mo^4+^	Mo^5+^	Mo^6+^
CoMo/γ‐Al_2_O_3_	229.2	230.5	232.5	232.1	233.9	236.7
CoMo‐EDTA/ γ‐Al_2_O_3_	229.1	230.0	232.0	232.0	233.6	234.5
CoMo‐AA/ γ‐Al_2_O_3_	227.0	230.4	232.4	228.7	233.7	236.4
CoMo‐CAA/ γ‐Al_2_O_3_	227.1	230.0	231.8	230.6	231.8	234.8

**Table 6:** XPS parameters of the different contributions of Co 2p obtained for unchelated and chelated CoMo/γ‐Al_2_O_3_ catalysts.

Catalysts	BE (eV) Co 2p_3/2_			BE (eV) Co 2p_1/2_		
	Co_9_S_8_	CoMoS	Co^2+^	Co_9_S_8_	CoMoS	Co^2+^
CoMo/ γ‐Al_2_O_3_	779.3	777.5	786.0	791.4	794.4	798.2
CoMo‐EDTA/ γ‐Al_2_O_3_	770.5	779.6	783.3	791.2	794.3	797.9
CoMo‐AA/ γ‐Al_2_O_3_	776.5	779.0	782.3	791.5	793.8	795.4
CoMo‐CA/ γ‐Al_2_O_3_	787.6	779.0	781.6	‐	783.7	797.8

**Table 7:** Percentage abundance of Co 2p and Mo 3d for CoMo/Al_2_O_3_ and CoMo‐EDTA/γ‐Al_2_O_3_ catalysts.

Catalysts	Co distribution (%)			Mo distribution (%)		
	Co_9_S_8_	CoMoS	Co^2+^	Mo^4+^	Mo^5+^	Mo^6+^
CoMo/ γ‐Al_2_O_3_	37	45	18	45	48	7
CoMo‐EDTA/ γ‐Al_2_O_3_	17	68	14	63	21	16
CoMo‐AA/ γ‐Al_2_O_3_	22	71	7	75	17	8
CoMo‐CA/ γ‐Al_2_O_3_	8	78	14	77	12	14

HRTEM measurements of sulfided catalysts were performed to gain more insight in the morphology and dispersion of active MoS_2_ crystallites induced by chelating ligands loading. The arrow shows the presence of Mo and Co metals present on the surface. Figure 6i (a, b, c, d) show typical fringes representing MoS_2_ crystallites.^[26]^ The morphology of MoS_2_ crystallites was modified by the DTA, AA, and CA. It is clear from (Figure 6) that the incorporation of chelating ligands had a significant impact on the aggregation of nanoparticles. The addition of chelating ligands led to agglomeration of nanoparticles, better dispersion, and smaller particle size (Table 3). Of interest is the presence of this interface which is beneficial for HDS formation.^[76]^ The catalysts, (CoMo/γ‐Al_2_O_3_, CoMo‐EDTA/γ‐Al_2_O_3_, CoMo‐AA/γ‐Al_2_O_3_ and CoMo‐CA/γ‐Al_2_O_3_) shows multilayer structure with lattice spacing ranging 0.63–0.65 nm, corresponding to the (0 0 2) plane of the MoS_2_ phase.[^44^, ^73^]

**Figure 7:**
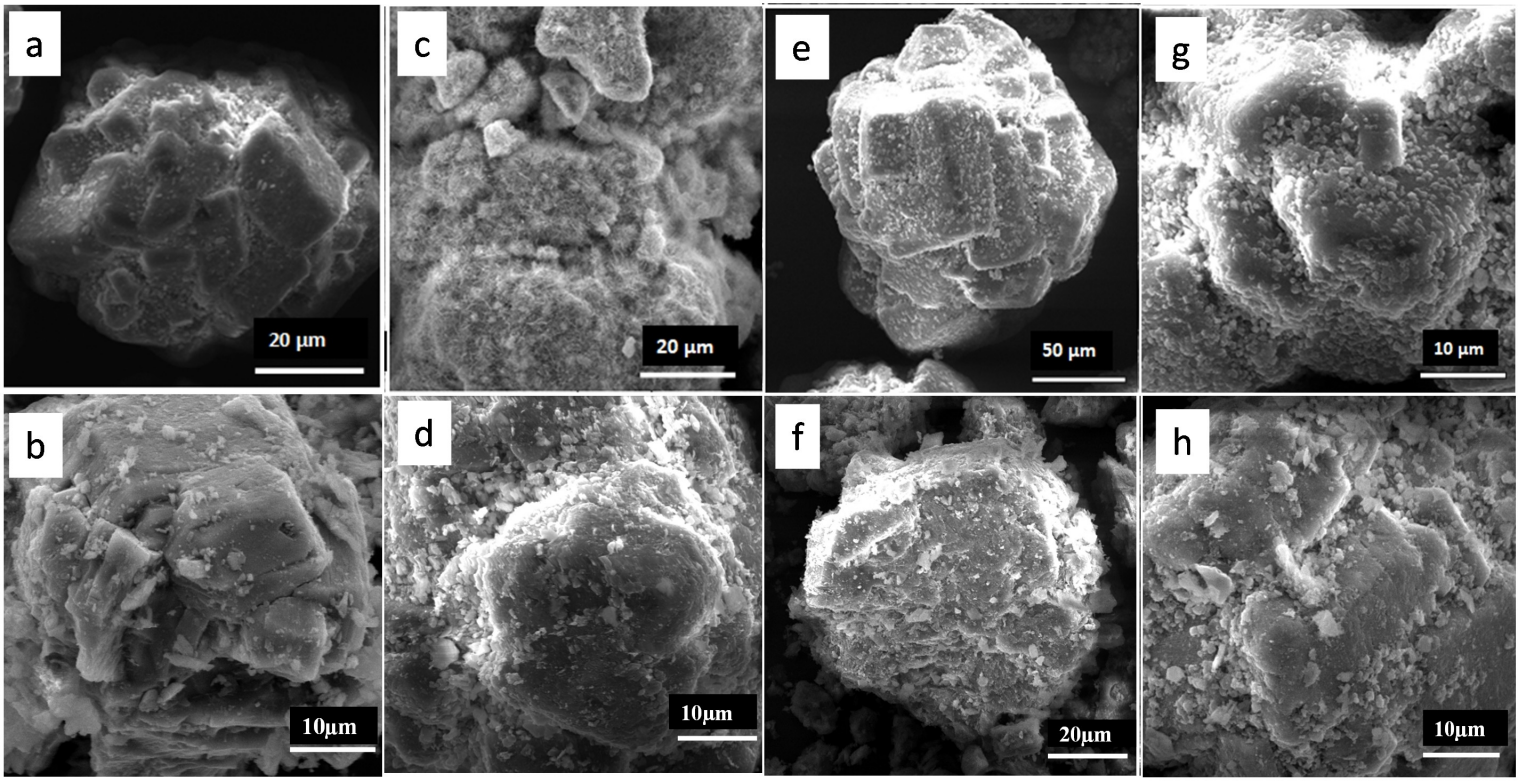
SEM images for CoMo/γ‐Al_2_O_3_ (a) oxide, (b) sulfided; CoMo‐EDTA/γ‐Al_2_O_3_ (c) oxide, (d) sulfided; CoMo‐AA/γ‐Al_2_O_3_ (e) oxide, (f) sulfided; CoMo‐CA/γ‐Al_2_O_3_ (g) oxide, (h) sulfided catalysts at different magnifications.

The SEM images of CoMoO_x_ are presented in Figure 7a, c, e, g. CoMo/γ‐Al_2_O_3_ showed spherical‐like particles, which are agglomerated with small particles present on the surface of the support (Figure 7a, c, e, g). CoMo‐EDTA/γ‐Al_2_O_3_ (Figure 7c) shows irregular shaped crystallites with small needle‐like particles that are highly agglomerated, as a result the average size distribution could not be measured. CoMo/γ‐Al_2_O_3_, CoMo‐AA/γ‐Al_2_O_3_ and CoMo‐CA/γ‐Al_2_O_3_ images (Figure 7a, e, and g) revealed agglomerated spherical‐like material with average size 107–133 μm. The sulfided CoMo catalysts are displayed in Figure 7b, d, f, h. The SEM images confirmed agglomerated particles with small fluffy like particles, which could indicate that the catalysts are porous in nature. Particle distributions was in the range of 98.3–104.8 μm (in diameter).

**Figure 8:**
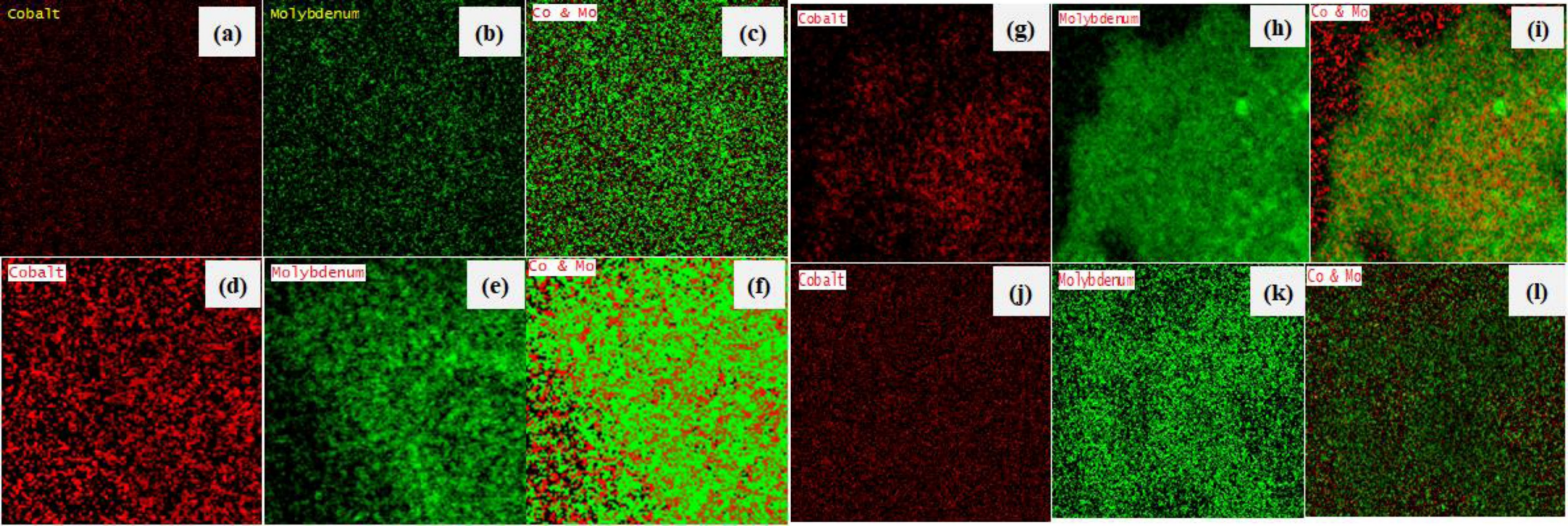
Elemental analysis images for (a‐c) CoMo/γ‐Al_2_O_3_, (d‐f) CoMo‐EDTA/γ‐Al_2_O_3_, (g‐i) CoMo‐AA/γ‐Al_2_O_3_, and (j‐l) CoMo‐CA/γ‐Al_2_O_3_.

EDS analysis indicated that S/Mo atomic ratio for all samples is around two (2.06–2.58), which is consistent with a MoS_2_ phase (Table S7). High amounts of carbon (25.450≤C/Mo≤37.50) are found in all the MoS_2_ catalysts (Figure S8). The main source of the carbon signal is most probably the heptane solvent or/and the tape used to hold the powders on the grid during the analysis.[^80^, ^81^] Elemental mapping analysis confirmed uniform dispersion of Co and Mo in all catalysts (Figure 8(a–l).[^75^, ^82^]

**Figure 9:**
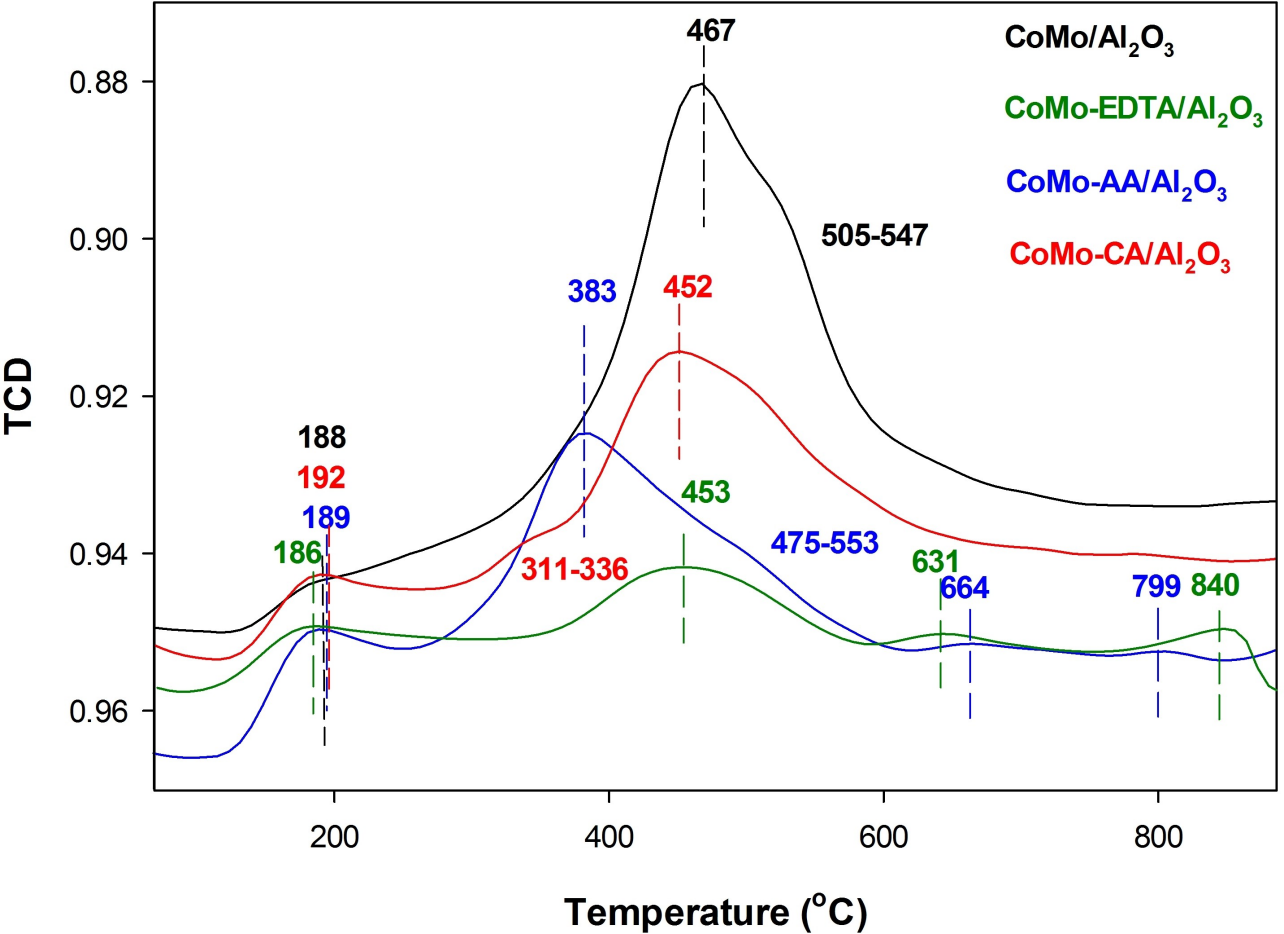
H_2_‐TPR analysis for sulfided CoMo/γ‐Al_2_O_3_, CoMo‐EDTA/γ‐Al_2_O_3_, CoMo‐AA/γ‐Al_2_O_3_, and CoMo‐CA/γ‐Al_2_O_3_ catalysts.

H_2_‐TPR analysis for sulfided CoMo catalysts was conducted to obtain information about the interaction between active sites and the support, and the metal‐sulfur interaction.[^39^, ^66^, ^83^, ^84^] Figure 9 shows the H_2_‐TPR−S profiles for CoMo/γ‐Al_2_O_3_, CoMo‐EDTA/γ‐Al_2_O_3_, CoMo‐AA/γ‐Al_2_O_3_, and CoMo‐CA/γ‐Al_2_O_3_ catalysts. These catalysts exhibit two defined reduction peaks. The reduction peaks at low‐temperature (188, 186, 189, and 192 °C) are ascribed to the reduction of sulfur atoms that are weakly bonded to the catalyst surface especially the edge S_2_^2−^ or sulfur bridges on MoS_2_, being associated with the coordinatively unsaturated sites (CUS).[^83^, ^84^, ^85^, ^86^, ^87^, ^88^, ^89^, ^90^] The high‐temperature reduction peaks for CoMo/γ‐Al_2_O_3_ (467 °C), CoMo‐EDTA/γ‐Al_2_O_3_ (453 °C), CoMo‐AA/γ‐Al_2_O_3_ (383 °C), and CoMo‐CA/γ‐Al_2_O_3_ (452 °C) are assigned to the reduction of labile sulfur species at the surface of MoS_2_ crystallites and the partial reduction of MoS_2_.[^59^, ^85^, ^86^, ^87^, ^88^, ^89^] The low temperature of CoMo‐AA/γ‐Al_2_O_3_ suggest an increase in reducibility of Mo species when acetic acid was used.[^59^, ^66^, ^91^] Generally, Co−Mo interaction on supported material occurs at similar temperatures as those assigned for the reduction of free Mo species, the data confirms varying degree of Co−Mo interaction. The shoulder peaks observed at 505–547 °C and 475–553 °C for CoMo/γ‐Al_2_O_3_ and CoMo‐AA/γ‐Al_2_O_3_ could be due to the partial reduction of β‐CoMoO_4_ to Co_2_Mo_3_O_8_ phase.^[84]^ The peaks at 631 °C and 840 °C for CoMo‐EDTA/γ‐Al_2_O_3_, as well as the peaks at 664 °C and 799 °C for CoMo‐AA/γ‐Al_2_O_3_, are associated with the reduction of the bulk MoS_2_ phase, which does not participate in hydrodesulfurization (HDS) reactions.^[86]^ The incorporation of chelating ligands drove the shift of peak position to lower temperatures, indicating the enhanced reducibility of MoS_2_. The MS signals for sulfided catalysts is shown in Figure S9.

Thermal behaviour (TGA and DSC) of the catalysts (a) CoMo/γ‐Al_2_O_3_ catalyst; (b) CoMo‐EDTA/γ‐Al_2_O_3_; (c) CoMo‐EDTA crystals; (d) CoMo‐AA/γ‐Al_2_O_3_; (e) CoMo‐AA crystal, and (f) CoMo‐CA/γ‐Al_2_O_3_) was studied in the range of 50–850 °C in N_2_ atmosphere (Figure S10a–f). The decomposition profiles are displayed in Table 8. Briefly, the thermal behavior showed weight loss in the temperature range of 100–150 °C with endothermic peaks around 80–180 °C and exothermic peaks around 120–210 °C (Table 8 and Figure S10a–e), due to the loss of adsorbed water and partial decomposition of the complex.[^10^, ^93^] A weight loss between 220–410 °C accompanied by exothermic between 200 °C and 350–450 °C is observed and is due to complex overlapping chemical reactions such as dihydroxylation of alumina, partial dehydration‐decomposition of metal precursor salts, and decomposition of the residual organic matrix.[^10^, ^17^, ^79^, ^93^, ^94^] Endothermic peaks were observed around 210–620 °C, due to partial hydration‐decomposition of the metal precursor salts, decomposition of the remaining organic residuals from chelating ligands, and further combustion of residual organic matrix, respectively.[^17^, ^93^] From 650 °C no weight loss was observed, this indicates a subsequent total transformation of the Co and Mo precursor species into the catalyst oxidic precursor.[^93^, ^94^] The weak exothermic peak at 600–860 °C was associated with the generation of MoO_3_ and formation of stable metal oxides.[^6^, ^42^, ^44^]

**Table 8:** Thermal decomposition data of complexes (**1–4**).

Complexes	Ranges of decomposition (°C)	Weight loss, % Found	Exothermic/ endothermic (°C)	Proposed reason(s) for the wt. loss/Exo and Endo processes
CoMo/γ‐Al_2_O_3_	<150	4.5	Endo (<150) Exo (160‐210)	Desorption of physically adsorbed water.^[93]^
	180–410	2.5	Exo (170‐360)	Partial dehydration of Co and Mo nitrate.^[92]^
	410–650	‐	Endo (400‐780)	Combustion of organic matrix and the formation of stable metal oxides.[^93^, ^94^]
CoMo‐EDTA/ γ‐Al_2_O_3_	<150	3.52	‐	Desorption of physically adsorbed water.
	150–220	3.4	Endo (180)	Desorption of physically adsorbed water.
	220–410	4.0	Exo (210,380, 410) Endo (240)	Partial dehyroxylation‐decomposition of EDTA^[17]^
	>600	‐	Exo (600‐800)	Formation of stable metal oxides.^[6]^
CoMo‐EDTA complex	<150	7.1	Endo (120)	Desorption of physically adsorbed water.^[10]^
	150–190	13.5	Endo (170)	Partial decomposition of EDTA.^[63]^
	250– 390	21.0	Exo (250, 340) Endo (280, 390)	Full decomposition of EDTA, and transformation of Co and Mo precursors species^[17]^
CoMo‐AA/ γ‐Al_2_O_3_	80–180	3.1	Exo (185)	Desorption of water and partial decomposition of acetic acid.^[94]^
	190–400	1.2	Endo (210‐620)	Dehydroxylation and decomposition of carbon residua.[^10^, ^94^]
	>400	‐	Endo (860)	Formation of stable metal oxides.^[48]^
CoMo‐AA complex	≤150	31	Endo (150)	Desorption of physically adsorbed water.
	250–380	22	Endo (240, 380) Exo (200, 270)	Partial decomposition of AA, transformation of metal species and the formation of stable metal oxide.^[44]^
CoMo‐CA/ γ‐Al_2_O_3_	50–180	1.1	Exo (80)	Desorption of water molecules^[52]^
	210–290	15.5	Exo (200)	complex series of overlapping chemical,[^51^, ^95^, ^96^] and full decomposition of CA.[^17^, ^52^]
	300–600	2.8	Exo (350–450)	evolution of remaining residue of citric acid.^[96]^
	560–770	‐	Exo (560–650, 770)	Formation of stable metal oxides [42].

### 2.2 Catalytic Activity

**Scheme 1:**
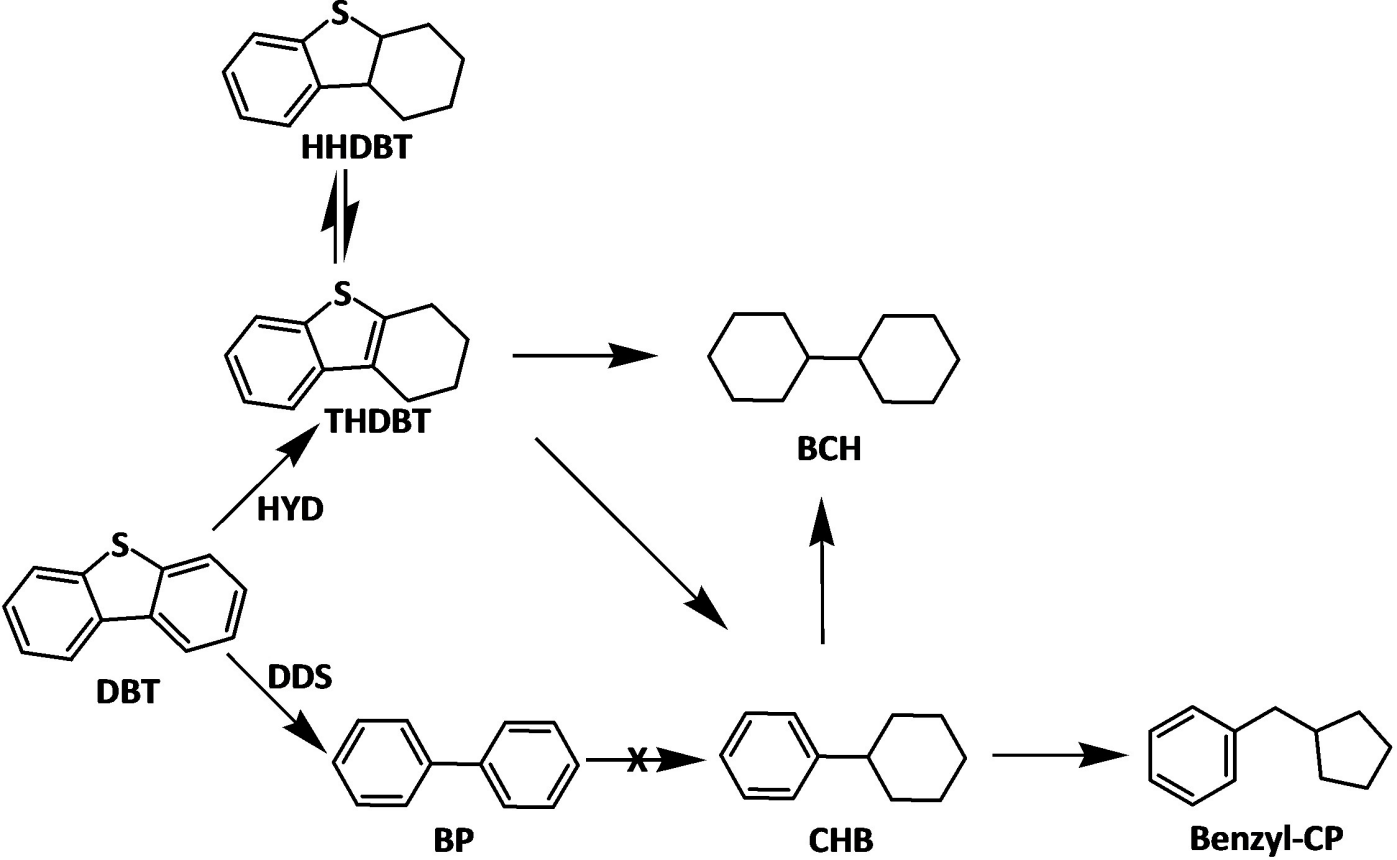
Proposed HDS Reaction Pathway of DBT.

The catalytic behaviour of CoMo/γ‐Al_2_O_3_, CoMo‐EDTA/γ‐Al_2_O_3_, CoMo‐AA/γ‐Al_2_O_3_, and CoMo‐CA/γ‐Al_2_O_3_ were tested in HDS of DBT (Figure S11). As shown in Table 9, HDS of DBT occurs via two routes the direct desulfurization (DDS) route and the hydrogenation (HYD) route (Scheme 1): direct desulfurization (DDS) leads to the formation of biphenyl (BP) and a hydrogenation (HYD) route which leads to the formation of tetrahydrodibenzothiophene (TH‐DBT), cyclohexylbenzene (CHB), bicyclohexyl (BCH) and benzylcyclopentane (benzyl‐CP).[^40^, ^98^, ^99^, ^100^, ^101^, ^102^] The increase in BP selectivity and a decrease in CHB selectivity in all catalysts was evidence that the DBT desulfurization degree was mostly *via* the DDS pathway. All the investigated catalysts achieved 43–98 % conversion of DBT. The maximal DBT conversion was observed for CoMo‐CA/γ‐Al_2_O_3_ (98 %), and CoMo/γ‐Al_2_O_3_ catalyst has the lowest activity with the conversion of 43 %. The conversion follows the order: CoMo‐CA/γ‐Al_2_O_3_ > CoMo‐AA/γ‐Al_2_O_3_ > CoMo‐EDTA/γ‐Al_2_O_3_ > CoMo/γ‐Al_2_O_3_. The obtained results are presented in Table 9 and for all the catalysts the selectivity favours the DDS pathway.

**Table 9:** Catalytic performances of CoMo/γ‐Al_2_O_3_ and CoMo‐x/γ‐Al_2_O_3_ (x=EDTA, AA, CA) in hydrotreating of DBT as a simulated fuel.

Catalysts	HDS (%)	BP	PhCh	HYD/DDS ratio	TOF (h^−1^)
CoMo/γ‐Al_2_O_3_	43	21	4	0.19	31
CoMo‐EDTA/γ‐Al_2_O_3_	90	63	23	0.37	56
CoMo‐AA/γ‐Al_2_O_3_	94	71	27	0.38	84
CoMo‐CA/γ‐Al_2_O_3_	98	76	18	0.24	150

Catalyst (Molybdenum content) employed =0.1 g (1.66×10^−5^ moles). Time of reaction for each of the oxidation system was 6 h. ^a^TOF, h^−1^: (turnover frequency) moles of substrate converted per mole of metal ion (in the solid‐state catalyst) per hour.

Activity was mainly influenced by a combined effect of (i) Co loading which induces the formation of Co_9_S_8_ over the CoMoS phase, (ii) Mo loading which influences the formation of hardly reduced MoO_3_ and difficult to sulfide and (iii) degree of dispersion (slab diameter) and reducibility of active sites, MoS_2_.[^75^, ^97^] However, in this study, the higher activity (with TOF numbers) is largely related to the degree of Mo content. Furthermore, data obtained from TPR further showed that MoS_2_ phases in CoMo‐AA/γ‐Al_2_O_3_ and CoMo‐CA/γ‐Al_2_O_3_ are easily reduced (when accessing labile sulfur species at the surface of MoS_2_ crystallites and the partial reduction of MoS_2_ are considered) compared to CoMo‐EDTA/γ‐Al_2_O_3_ and CoMo/γ‐Al_2_O_3._ The HDS product distribution of DBT are illustrated in **F**igures S12 and S13.

Additionally, the stability of catalysts was assessed as a crucial indicator for practical applications.^[103]^ CoMo‐CA/γ‐Al_2_O_3_, CoMo‐AA/γ‐Al_2_O_3_, CoMo‐EDTA/γ‐Al_2_O_3_, and CoMo/γ‐Al_2_O_3_ were reused in the HDS of DBT for two cycles continuously, and the results are summarized in Figure S14, which indicates the good stability of CoMo‐CA/γ‐Al_2_O_3_ and CoMo‐AA/γ‐Al_2_O_3_ during DBT HDS. Thus, the outstanding hydrodesulfurization (HDS) activity and stability of our catalyst indicate promising potential for industrial applications.

Catalysts were analysed by means of EDS spectroscopy to quantify the elemental composition of CoMo/γ‐Al_2_O_3_ catalyst surface. This included measuring the concentrations of key components such as cobalt (Co), molybdenum (Mo), sulfur (S), and support materials (Al) (Table S8, Figure S15). It was observed after three continuous HDS cycles we observed a significant reduction in amount of Co and Mo across CoMo‐CA/γ‐Al_2_O_3_, CoMo‐EDTA/γ‐Al_2_O_3_ and CoMo/γ‐Al_2_O_3_ except for CoMo‐AA/γ‐Al_2_O_3_ which leached little quantities of Co and Mo compared to the other catalysts (Table S8). The results further demonstrated that catalyst maintained its active phase ratio corresponding to MoS_2_ except for CoMo‐EDTA/ γ‐Al_2_O_3_ and CoMo/γ‐Al_2_O_3_ (MoO_3_) which could indicate catalyst deactivation mechanisms, such as sintering, poisoning, or phase transformation.

## Conclusions

In the present work, CoMo‐based catalysts (CoMo/γ‐Al_2_O_3_, CoMo‐EDTA/γ‐Al_2_O_3_, CoMo‐AA/γ‐Al_2_O_3_, CoMo‐CA/γ‐Al_2_O_3_) were successfully synthesized by hydrothermal method, and the catalysts were evaluated in the HDS of DBT. The catalytic results confirm that the introduction of ligand to CoMo/γ‐Al_2_O_3_ plays an important role in the HDS performance. The CoMo‐CA/γ‐Al_2_O_3_ catalyst showed the highest catalytic performance among all the investigated catalysts, whereas CoMo‐AA/γ‐Al_2_O_3_ presented the highest HYD/DDS ratio of 0.38. The latter is directly linked to a higher pore size distribution pattern of CoMo‐AA/γ‐Al_2_O_3_ which enhances the accessibility of dibenzothiophene (DBT) to the catalyst's active sites, thus affecting its selectivity and overall catalytic activity. The presence of AA in CoMo‐AA/γ‐Al_2_O_3_ weaken the metal‐support interactions as observed in H_2_‐TPR data during the reduction of labile sulfur species of MoS_2_. The slight decrease in reduction temperature of active metals (MoS_2_) and enhanced catalyst surface properties promoted the formation of “Type II” Co−Mo−S phases, favouring planar DBT adsorption and improving HDS performance. The presented results open a new viewpoint for the use of relatively weak bidentate ligands as possible chelating agents for HDS catalyst synthesis.

## Crystal Data

Deposition Number(s) 2085133 (for CoMo‐AA(a)), 2085135 (for CoMo‐AA(b)), 2085134 (for CoMo‐EDTA) contain(s) the supplementary crystallographic data for this paper. These data are provided free of charge by the joint Cambridge Crystallographic Data Centre and Fachinformationszentrum Karlsruhe Access Structures service.

## Conflict of Interests

The authors declare no conflict of interests.

3

## Supporting information

As a service to our authors and readers, this journal provides supporting information supplied by the authors. Such materials are peer reviewed and may be re‐organized for online delivery, but are not copy‐edited or typeset. Technical support issues arising from supporting information (other than missing files) should be addressed to the authors.

Supporting Information

## Data Availability

The data that support the findings of this study are available in the supplementary material of this article.

## References

[1] T. A. Saleh, *Applying Nanotechnology to the Desulfurization Process in Petroleum Engineering*, IGI Global, Hershey, Pennsylvania, USA **2015**.

[2] Y. Okamoto , et al., J. Catal. 2009, 265(2), 216–228.

[3] M. S. Rana , et al., J. Catal. 2007, 246(1), 100–108.

[4] N.Sareen, S.Bhattacharya, *Applying Nanotechnology to the Desulfurization Process in Petroleum Engineering*, IGI Global, Hershey, Pennsylvania, USA **2016**, pp. 84–128.

[5] H. Topsøe , et al., J. Catal. 1981, 68(2), 433–452.

[6] S. Badoga , et al., Appl. Catal. B: Environ. 2012, 125, 67–84.

[7] L. Qu , W. Zhang , P. J. Kooyman , R. Prins , J. Catal. 2003, 215(1), 7–13.

[8] J. Chen , et al., Ind. Eng. Chem. Res. 2017, 56(48), 14172–14181.

[9] K. Al-Dalama , A. Stanislaus , Energy Fuels 2006, 20(5), 1777–1783.

[10] N. Q. Bui , C. Geantet , G. Berhault , Appl Catal. A: Gen. 2019, 572, 185–96.

[11] L. Peña , D. Valencia , T. Klimova , Appl Catal., B. 2014, 147, 879–87.

[12] P. A. Nikulshin , D. I. Ishutenko , A. A. Mozhaev , K. I. Maslakov , A. A. Pimerzin , J. Catal. 2014, 312, 152–169.

[13] V. Sundaramurthy , A. K. Dalai , J. Adjaye , Catal. Lett. 2005, 102, 299–306.

[14] A. I. Dugulan , M. W. Crajé , G. J. Kearley , J. Catal. 2004, 222(1), 281–284.

[15] P. Blanchard , et al., C R Chim. 2016, 19(10), 1286–1302.

[16] S. V. Budukva , et al., Catal. Today 2019, 329, 35–43.

[17] S. L. González-Cortés , et al., Appl. Petrochem. Res. 2015, 5, 181–197.

[18] D. Valencia , T. Klimova , Appl. Catal. B 2013, 129, 137–145.

[19] Y. Zhang , et al., Catal. Commun. 2016, 82, 20–33.

[20] J. N. Díaz de León , et al., Catalysts 2019, 9(1), 87.

[21] T. E. Klimova , et al., J. Catal. 2013, 304, 29–46.

[22] L. Van Haandel , G. M. Bremmer , E. J. Hensen , T. Weber , J. Catal. 2017, 351, 95–106.

[23] I. Shafiq , et al., Catal. Rev. 2022, 64(1), 1–86.

[24] G. M. Sheldrick , Acta Crystallogr. Sect. C: Struct. Chem. 2015, 71(1), 3–8. 25567568 10.1107/S2053229614024218 PMC4294323

[25] L. J. Farrugia , J. Appl. Crystallogr. 2012, 45(4), 849–854.

[26] Y. V. Vatutina , et al., Catal. Today 2019, 329, 13–23.

[27] A. Guha , et al., J. Mol. Catal. A Chem. 2011, 338(1–2), 51–57.

[28] A. K. Maji , et al., J. Mol. Struct. 2017, 1146, 821–827.

[29] R. Huirache-Acuña , et al., Fuel 2015, 149, 149–161.

[30] P. Castillo-Villalón , et al., Catal. Today 2016, 259, 140–149.

[31] J. A. Bergwerff , T. Visser , B. M. Weckhuysen , Catal. Today 2008, 130(1), 117–125.

[32] Y. V. Vatutina , et al., Catal. Today 2019, 329, 13–23.

[33] L. G. van de Water , et al., J. Catal. 2006, 242(2), 287–298.

[34] J. Chen , et al., Ind. Eng. Chem. Res. 2017, 56(48), 14172–14181.

[35] J. Vakros , et al., Appl. Catal. B Environ. 2010, 96(3–4), 496–507.

[36] N. Rinaldi , K. Al-Dalama , T. Kubota , Y. Okamoto , Appl. Catal. A Gen. 2009, 360(2), 130–136.

[37] R. S. Weber , J. Catal. 1995, 151(2), 470–474.

[38] C. Zhang , et al., Appl. Catal. A Gen. 2019, 570, 84–95.

[39] C. Zhang , et al., Appl. Catal. A Gen. 2019, 575, 187–197.

[40] R. Obeso-Estrella , et al., Fuel 2018, 233, 644–657.

[41] C. I. Cabello , et al., J. Mol. Catal. A Chem. 2002, 186(1–2), 89–100.

[42] S. Wang , et al., Sci. Rep. 2018, 8(1), 3182. 29453375 10.1038/s41598-018-21436-4 PMC5816628

[43] F. J. Méndez , et al., Appl. Catal. B Environ. 2017, 219, 479–491.

[44] H. Wu , et al., Fuel 2014, 130, 203–210.

[45] M. O. Kazakov , et al., Catal. Today 2019, 329, 108–115.

[46] H. Tian , C. A. Roberts , I. E. Wachs , J. Phys. Chem. C 2010, 114(33), 14110–14120.

[47] S. Shan , et al., J. Catal. 2015, 330, 288–301.

[48] R. Cattaneo , T. Shido , R. Prins , J. Catal. 1999, 185(1), 199–212.

[49] J. Xu , T. Huang , Y. Fan , Appl. Catal. B Environ. 2017, 203, 839–850.

[50] O. V. Klimov , et al., J. Mol. Catal. A Chem. 2010, 322(1–2), 80–89.

[51] J. Escobar , et al., Fuel Process. Technol. 2017, 156, 33–42.

[52] D. Zhang , et al., J. Chem. Eng. 2017, 330, 706–717.

[53] F. Rashidi , et al., J. Catal. 2013, 299, 321–335.

[54] Q. Wei , et al., J. Am. Chem. Soc. 2011, 3–13.22191666

[55] Y. Fan , et al., J. Catal. 2011, 279(1), 27–35.

[56] X. Wang , et al., Energy Fuels 2018, 32(7), 7800–7809.

[57] S. Jiang , et al., RSC Adv. 2016, 6(108), 106680–106689.

[58] K. A. Nadeina , et al., Catal. Today 2019, 329, 2–12.

[59] B. Liu , et al., Appl. Catal. A Gen. 2014, 471, 70–79.

[60] W. Trakarnpruk , B. Seentrakoon , Ind. Eng. Chem. Res. 2007, 46(7), 1874–1882.

[61] M. Rudolph , M. Motylenko , D. Rafaja , IUCr J. 2019, 6(1), 116–127. 10.1107/S2052252518015786 PMC6327185 30713709

[62] Y. Zhang , et al., Catal. Commun. 2016, 82, 20–23.

[63] M. Nikulshina , et al., Catal. Today 2019, 329, 24–34.

[64] M. A. Pérez-Sosa , et al., Int. J. Hydrogen Energy 2021, 46(41), 21419–21432.

[65] Q. Liu , et al., Rare Met. 2019, 38, 1–3.

[66] N. Frizi , et al., Catal. Today 2008, 130(2–4), 272–282.

[67] A. D. Gandubert , et al., Oil Gas Sci. Technol. 2007, 62(1), 79–89.

[68] O. V. Klimov , et al., Catal. Today 2018, 307, 73–83.

[69] T. K. Ninh , L. Massin , D. Laurenti , M. Vrinat , Appl. Catal. A Gen. 2011, 407(1–2), 29–39.

[70] P. A. Nikulshin , et al., Appl. Catal. B Environ. 2014, 158, 161–174.

[71] J. C. Dupin , et al., Thin Solid Films 2001, 384(1), 23–32.

[72] C. Contreras , et al., J. Nanotechnol. 2016, 2016, 3752484.

[73] T. Huang , J. Xu , Y. Fan , Appl. Catal. B Environ. 2018, 220, 42–56.

[74] Y. Muhammad , et al., RSC Adv. 2019, 9(18), 10371–10385. 35520937 10.1039/c9ra00095j PMC9062605

[75] L. Peña , D. Valencia , T. Klimova , Appl. Catal. B Environ. 2014, 147, 879–887.

[76] X. Xi , et al., Appl. Catal. B Environ. 2020, 272, 118950.

[77] J. Whelan , et al., Energ Fuels 2018, 32(7), 7820–7826.

[78] L. A. Zavala-Sanchez , et al., ACS Catal. 2020, 10(11), 6568–6578.

[79] W. Song , et al., ACS Catal. 2018, 9(1), 259–268.

[80] M. Rudolph , M. Motylenko , D. Rafaja , IUCr J. 2019, 6(1), 116–127. 10.1107/S2052252518015786 PMC6327185 30713709

[81] Y. Zhang , et al., Catal. Commun. 2016, 82, 20–23.

[82] J. Cortez-Elizalde , et al., Catal. Today 2022, 392, 116–130.

[83] Y. Xu , et al., J. Catal. 2022, 407, 19–28.

[84] A. Kokliukhin , et al., Catal. Today 2021, 377, 26–37.

[85] H. Liu , et al., Appl. Catal. B Environ. 2015, 174, 264–276.

[86] F. A. Braggio , et al., Catal. Lett. 2017, 147, 1104–1113.

[87] B. Liu , et al., Fuel 2018, 234, 1144–1153.

[88] B. Liu , et al., Ind. Eng. Chem. Res. 2018, 57(6), 2041–2049.

[89] L. I. Bin , et al., J. Fuel Chem. Technol. 2018, 46(4), 441–450.

[90] J. Xu , et al., Appl. Catal. B Environ. 2019, 244, 385–395.

[91] L. I. Juan , et al., J. Fuel Chem. Technol. 2021, 49(10), 1513–1521.

[92] L. Zavala-Sanchez , et al., Nanotechnology 2019, 31(3), 035706. 31557737 10.1088/1361-6528/ab483c

[93] S. L. González-Cortés , et al., J. Mol. Catal. A Chem. 2005, 240(1–2), 214–225.

[94] K. Al-Dalama , A. Stanislaus , Thermochim. Acta. 2011, 520(1–2), 67–74.

[95] H. Knözinger , P. Ratnasamy , Catal. Rev. Sci. Eng. 1978, 17(1), 31–70.

[96] Z. D. Huang , et al., Catal. Lett. 2008, 122, 57–67.

[97] C. Yang , et al., Chin. J. Catal. 2023, 46, 125–136.

[98] P. G. Moses , et al., J. Catal. 2007, 248(2), 188–203.

[99] F. Bataille , et al., J. Catal. 2000, 191(2), 409–422.

[100] G. Zhang , et al., Fuel 2022, 322, 124160.

[101] L. Li , M. Wang , L. Huang , X. Liu , X. Zhang , H. Sun , Q. Yu , F. Yang , Q. Guo , B. Shen , Appl. Catal., B 2019, 254, 360–370.

[102] L. B. Romero-Sánchez , G. Alonso-Núñez , R. Prieto-García , et al., React. Kinet. Mech. Cat. 2021, 133, 1027–1044. 10.1007/s11144-021-02040-6.

[103] X. Kang , D. Wang , J. Liu , C. Tian , H. Xu , J. Xu , H. Fu , J. Mater. Chem. A. 2022, 10, 7263–7270.

