# Supplementary material for: Influence of Ligands on the Surface Characteristics of CoMo/γ‐Al2O3 and Hydrodesulfurization Catalytic Activity on Dibenzothiophene‐Type Compounds
Source: ChemistryOpen. 2025 Mar 3;14(4):e202400123. doi: 10.1002/open.202400123 (PMC11973508; doi:10.1002/open.202400123)
Supplement: Supplementary file 1 — Supporting Information [file OPEN-14-e202400123-s001.pdf]

ChemistryOpen

Supporting Information

Influence of Ligands on the Surface Characteristics of CoMo/γ-Al_2O_3 and Hydrodesulfurization Catalytic Activity on Dibenzothiophene-Type Compounds

Siphumelele Majodina,* Ryan Walmsley, Alisa Govender, Eric C. Hosten, Jaco Olivier, Zenixole Tshentu, and Adeniyi S. Ogunlaja*

SUPPLIMENTARY MATERIALS

Influence of Ligands on the Surface Characteristics of CoMo/γ-Al_2O_3 and Hydrodesulfurization Catalytic Activity on Dibenzothiophene-Type Compounds

Siphumelele Majodina[1*], Ryan Walmsley[2], Alisa Govender[3], Eric C. Hosten[1], Jaco Olivier[4], Zenixole Tshentu[1*], Adeniyi S. Ogunlaja[1*]

[1]Department of Chemistry, Nelson Mandela University, Gqeberha, South Africa, [2]Research and Development Division, Sasol Technology (Pty) Ltd, Sasolburg, South Africa [3]Materials Division, Sasol Technology (Pty) Ltd, Sasolburg, South Africa [4]Department of Physics and Centre for High Resolution (HRTEM), Nelson Mandela University, Gqeberha (Port Elizabeth), South Africa.

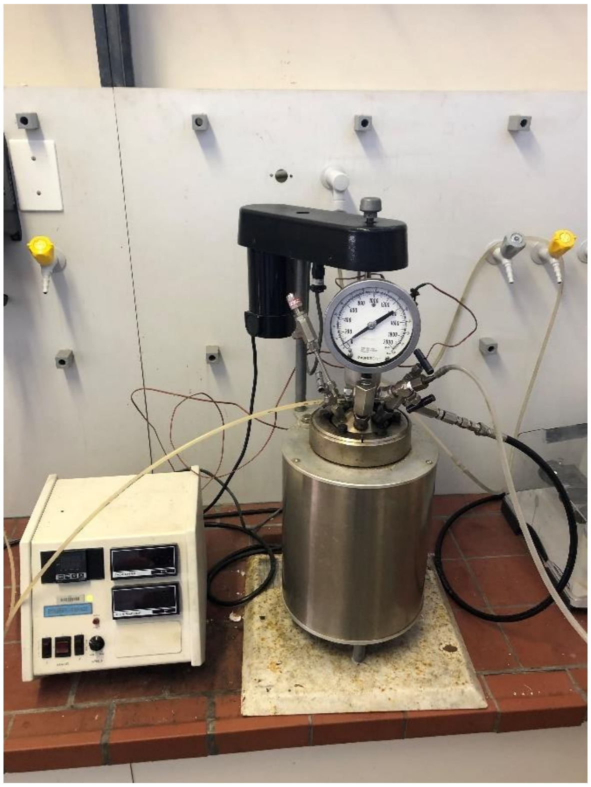

Figure S1: Hydrotreating reaction experiment setup for Parr reactor

S1 Characterization

using APEX2 data collection software for cell refinement and data reduction. Full detail of data collection using SHELXL-2018/3 [24] and ORTEP-3 for windows [25]. Data were corrected for absorption effects using the numerical method implemented in SADABS. The structures were solved by dual-space methods applying SHELXT-2018/2 and refined by least-squares procedures using SHELXL-2018/3 [24] with SHELXLE as a graphical interface. All non-hydrogen atoms were refined anisotropically, and the carbon-bound hydrogen atoms were added in idealized geometrical positions in a riding model. The crystal structure diagrams were drawn with ORTEP-3 for windows [25].

UV-vis spectra of studied samples were recorded on a Shimadzu UV vis-DRS Spectrophotometer UV-3100 with an MPCF-3100 sample compartment. with samples mounted between two quartz discs which fit into a sample holder coated with barium sulfate. The spectra were recorded over the wavelength range of 800-200 nm, and the scans were conducted at a medium speed using a 20 nm slit width.

The Tauc and Devis-Mott equation (1) was used to probe the optical band gap energy of catalysts from UV-Vis absorption spectroscopy. The equation is expressed as follows:

$$(\alpha h\nu)^{\frac{1}{n}} = A\,(h\nu - E_g) \qquad (1)$$

where α is the absorption coefficient, hν is the incident photon energy, A is the proportionality constant, E_g is the optical band gap energy, n represents the nature of electronic transition (n = 1/2 for direct transition).

The FT-IR spectra of solid samples were acquired in a single mode with a resolution of 4 cm-1, in the region from 4000 to 400 cm-1 and at an average of 32 scans using a Bruker Tensor 27 platinum ATR-FTIR spectrometer. Brunauer–Emmett–Teller (BET) Surface area and pore size distribution were measured using Micrometrics TriStar II 3020 Surface Area Analyzer. A Perkin Elmer SCIEX Elan-6100 ICP-OES AS-90 with auto sampler was used to determine the concentration of the sample. The Powder X-ray diffraction (PXRD) patterns of the powders were recorded by means of a Bruker D2 powder x-ray diffractometer using Cu radiation with a Lynxeye detector. A scan range of 5-70° 2Ө at 0.02o per step was used. All data analysis and Rietveld refinement were done using Topas® V6 software. Phase analysis was made with the use of diffraction database Powder Diffraction File (PDF).

TGA-DSC of solid samples were measured using a Perkin Elmer STA 6000 with a thermocouple sensor Pt-Pt/Rh, a crucible pan was used as a sample holder for all samples studied. A heating range was 55 to 900 °C with a flow rate of 30 mL min-1 under nitrogen and a constant heating rate of 20 °C/min was used for all the oxide samples studied.

EDS and SEM samples were obtained on a TESCAN Vegas TS 5136LM working at 20 kV. Sulfided catalysts were analyzed by XPS to identify the chemical species on the surface using Kratos Axis Ultra X-ray Photoelectron Spectrometer equipped with a monochromatic Al Kα source (1486.6 eV). The base pressure of the system was below 3 x 10-7 Pa. XPS experiments were recorded with a 75 W power source using hybrid-slot spectral acquisition mode and an angular acceptance angle of +/-20°. Kratos version 2 program was used for XPS data analysis and fitting carried out using a Gaussian line shape. Transmission electron microscopy (TEM) was performed using a double-aberration corrected JEOL JEM-ARM 200F (Jeol, Italy) operated at 200 kV and equipped with an Oxford Xmax 100 EDS detector and Gatan GIF 965ERS with dual electron-energy loss spectroscopy (EELS) capability. Imaging and analysis of the samples were done in TEM using parallel illumination and scanning mode (STEM), using a sub-angstrom sized probe with a probe current between 68 pA and 281 pA. The convergence semi-angle of the probe used was fixed at 23 mrad with acceptance semi-angles of the GIF and dark-field detector being 84 mrad and 34 to 137 mrad respectively. The BF detector acceptance semi-angle was set at 0 to 12 mrad by using an illumination limiting aperture. The EELS spectrum imaging was done using a 0.5 eV or 1 eV energy channel width for an energy range containing 2048 channels. The FWHM of the zero-loss peak was measured as 1.5 eV. EELS elemental distribution and compositional maps were generated using a model-based (Hartree-Slater) quantification routine (excluding ELNES). The selected area electron diffraction (SAED) was performed in TEM mode using parallel illumination. Recording of images was done using a GATAN Ultrascan SC1000 2K camera.

S2. Catalyst sulfidation and hydrodesulfurization measurements

Prior to the catalytic tests, the catalysts sample (2 g) were sulfided using 100 mL heptane solution-containing 10 wt.% of CS2 under hydrogen pressure of 4.0 MPa with the temperature of 573 K and maintained for 4 h to ensure complete sulfidation. The sulfided catalysts were recovered by filtration and dried. After sulfiding, HDS studies of the catalysts were performed in 100 mL of heptane containing (0.22 g) DBT under H2 pressure of 4.0 MP with 573 K and maintained for 6 h. The hydrotreating of model fuel was performed in a Parr reactor operating at typical industrial conditions (Fig. S1). The model fuel consisted of 374 ppmS in dibenzothiophene (DBT). The conversion of DBT and selectivity was determined using GC-MS. GC conditions for the determination of HDS products were carried out by using a ZB-5MSi capillary column (30 m x 0.25 mm x 0.25 μm). Helium was used as the carrier gas at a flow rate of 1.63 mL min-1 with an average velocity of 30.16 cm. sec-1 and a pressure of 63.73 KPa. The analysis run was started with an oven temperature of 40°C ramping to 300°C @ 15°C min^{-1}.

The HDS activity of the catalysts was estimated using equation 2: calculations are provided on the supplementary

$$X_{HDS}(\%) = \frac{C^0_{DBT} - C_{DBT}}{C^0_{DBT}} \times 100 \quad (2)$$

where C^0_{DBT} is the DBT content in the feedstock (wt.%) and C_{DBT} was the DBT content in the products (wt. %) [26]. The catalytic selectivity determined from the ratio between hydrogenation (HYD) and direct desulfurization (DDS) can be approximated by the following equation 3:

$$S_{HYD}/S_{DDS} = \frac{C_x}{C^0_{DBT} - C_{DBT}} \quad (3)$$

where C_x was the content of PhCH or BP [26].

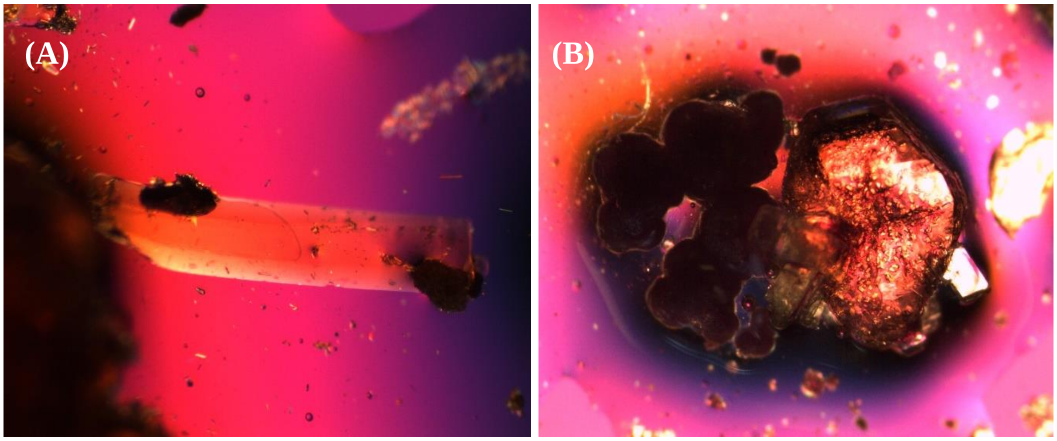

Figure S2: Images of crystals of (A) CoMo-AA and (B) CoMo-EDTA

S3 Single crystal description of CoMo-EDTA and CoMo-AA

The ORTEP diagram of the crystal structures of CoMo-EDTA and CoMo-AA are presented in **Fig. 1 & Fig. 2 (main manuscript)**. CoMo-EDTA crystallizes in the orthorhombic space group with Pmn21 (No.31). Co atom is coordinated with EDTA in a hepta coordinated–(N_2O_5) environment [26-28]. Bond angle data for Co and Mo as central atoms in CoMo-EDTA and Hydrogen bond parameters data are presented in **Table S2 and S3,** respectively. The coordination environment of Co(1) was formed by seven donors: five oxygen [O(1), O(1)i, O(3), O(3)i, O(5)] (i: 1-x, +y. +z) donor atoms, and two amine nitrogen's [N(1) and N(2)]. The presence of water molecule led to distortion, and the coordination of the donors led to an asymmetric dimer shown in Table S1. The Co(1)–O bond distances where the oxygen is connected with sodium ions was slightly different than that coordinated with acetate ion donors; Co(1)–O(1 &1i) = 2.142(4) Å, Co(1)–O(5) = 2.277(4) was slightly higher than that of Co(1)–O(3 & 3i) = 2.106(5) Å, and the coordination of Co(1)–N(1) = 2.280(5) Å and Co(1)–N(2) = 2.275(6) has longer bond lengths than the average oxygen donors [27]. This might be due to different steric effect and electron density around N and

Co atoms [26, 27]. Table S2 shows bond angles between the metal and donors. The degree of distortion in Co complex are reflected in the cisoid [74.50(15) –79.77(12) Å] and transoid angles [116.70(13) –148.02(4) Å] [28].

The presence of water molecules in the unit cell contribute to the distortion of the octahedral coordination sphere. In Mo (1-4)–O(8, 23, 13, 12, 16 & 17) the bond distances are in the range 1.709–1.723 Å and was significantly lower than that of Mo(1-4)–O(9, 10, 11, 14, 15, 17, 19 & 27) in the range of 1.934–2.502 Å (**Table S1**). This might be due to the bridging character of the oxides or higher charge density compared to water molecules [27]. The degree of distortion reflected in the *cisoid* for molybdenum; for Mo(1) [72.26(10) –104.00(16)^o], Mo(2) [69.71(7)–105.83(11)^o], Mo(3) [71.82(10)–105.15(16)^o] and Mo(4) [77.15(12)–171.77(9)]. While the *transoid* angles for molybdenum; Mo(1) [155.26(15)–162.98(14)^o], Mo(2) [146.36(14)–175.54(11)^o], Mo(3) [154.17(15)–163.42(14)^o] and Mo(4) [143.39(16) –171.77(9)^o], this deviated from linearity and this was likely due to the bridging features and steric requirement in the connection of the two units Bond angle data for Co and Mo as central atoms in CoMo-EDTA is presented in **Table S2**. Both Mo and Co complexes are connected by water molecule, where Co was connected via intramolecular H bond, while Mo was connected via oxygen of water lattice and Mo was also connected via oxygen of acetate ion from Co ion, respectively [27]. Hydrogen bond parameters for complex CoMo-EDTA crystal is presented in **Table S3**.

CoMo-AA crystallizes in the monoclinic space group with P21/c. The asymmetric unit comprises of one crystallographically independent Co(II) ions complex with acetic acid and water molecules, designated as Co(1) presenting distorted octahedral geometry. The coordination environment of Co(1) was formed by six donors: the equatorial plane constructed by four [O(3), O(3^i), O(4) and O(4^i)] (i: 1-x, 1-y, 1-z) donors atoms from water molecules while the axial position were occupied by the remaining two donor atoms originate from acetate ions [O(1) and O(1^i)], respectively [27, 28]. Selected bond lengths and angles pertaining to the coordination are tabulated in **Tables S4** and **Table S5**. The Co(1)-O bond distance of Co with the water molecules was slightly different with that of acetate ion donors; for Co(1)-O(1&1^i) = 2.0962(8) Å was slightly higher than that of Co(1)-O(3&3^i) = 2.0892(10) Å and, Co(1)- (4&4^i) = 2.1195(9) Å [34, 35]. This might be due to the rigidity of acetate ions or higher charge density compared to water molecules [27, 28]. The degrees of distortion of the coordination spheres are reflected in the *cisoid* [89.34(4)-90.57(4) ^{o}] and the *transoid* angles [28]. The sum (360°) of the equatorial angles [O(3)-Co(1)-O(4) 89.34(4)^o , O(3^i)-Co(1)-O(4) 90.66(4)^o , O(3^i)-Co(1)-O(4^i) 89.34(4)^o , O(3)-Co(1)-O(4^i) 90.66(4)^o was equivalent to 360°, this reflected that the equatorial oxygen's were in the same plane with the metal center [28]. The complex structure was stabilized by intramolecular H-bonds occurring between the H atoms of the four coordinated water molecules O(3), O(3^i) and O(4), O(4^i) with oxygen's O(1,2) and O(1^i, 2^i) from the acetate ion,

respectively [27, 28]. The crystal data of CoMo-AA(A), interact with each other via O—H…O hydrogen bonds (**Table S6**).

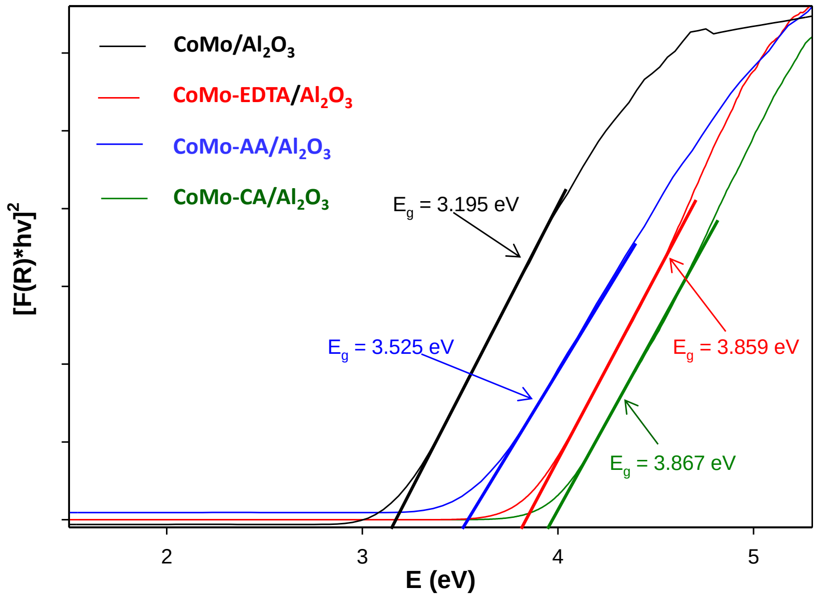

Figure S3: The Tauc plot for CoMo/γ-Al_2O_3, CoMo-x/γ-Al_2O_3 (x = EDTA, AA, CA) obtained from a UV-Vis spectrum.

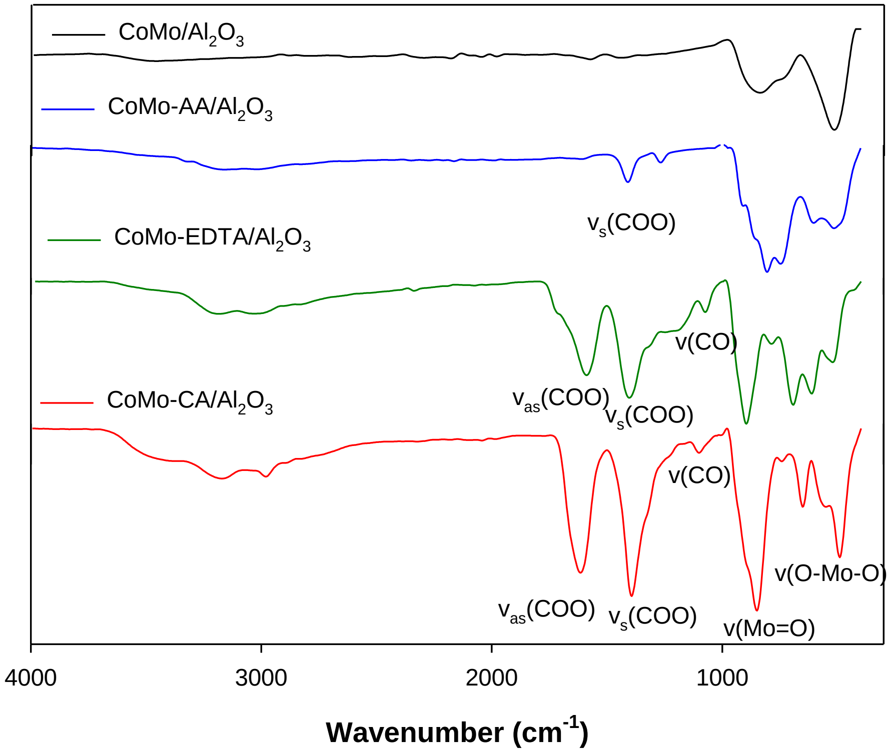

Figure S4: FT-IR spectra of CoMo/γ-Al_2O_3, CoMo-AA/γ-Al_2O_3, CoMo-EDTA/γ-Al_2O_3 and CoMo-CA/γ-Al_2O_3 catalyst.

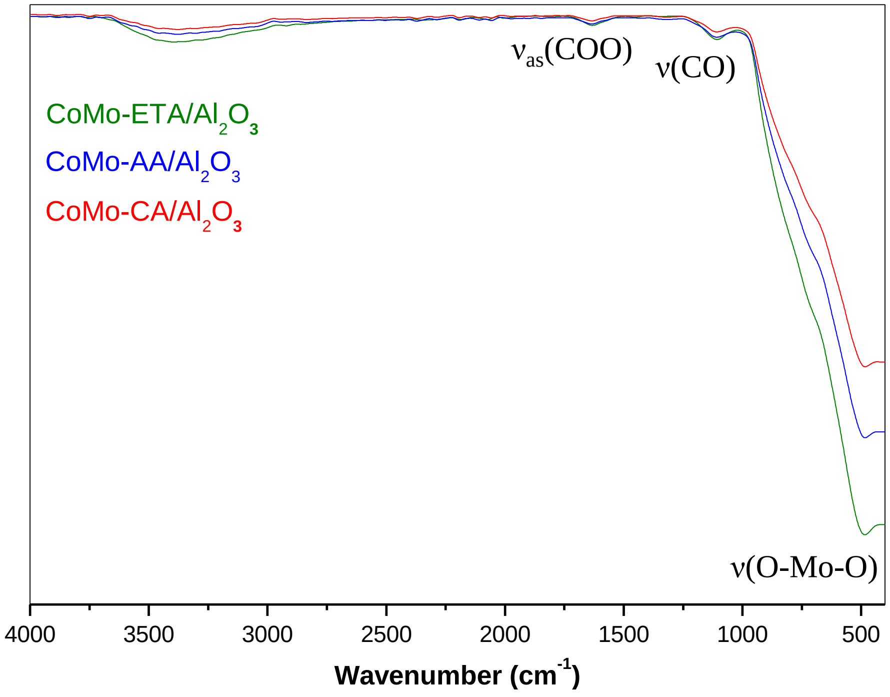

Figure S5: FT-IR spectra of sulfided CoMo/γ-Al_2O_3, CoMo-AA/γ-Al_2O_3, CoMo-EDTA/γ-Al_2O_3 and CoMo-CA/γ-Al_2O_3 catalyst.

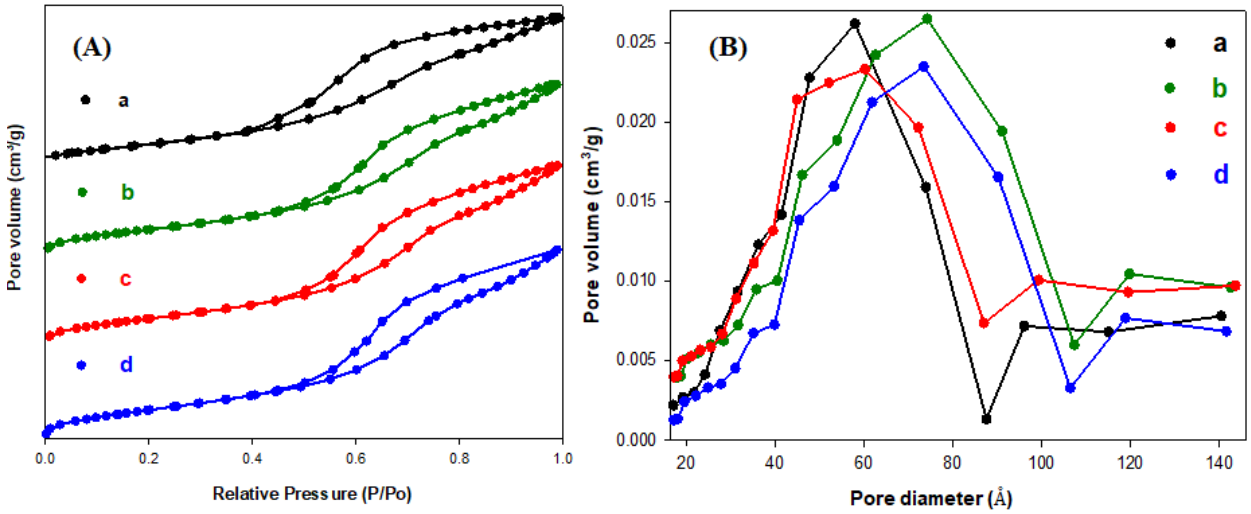

Figure S6: (A) N_2 adsorption-desorption isotherms and (B) pore size distribution patterns of series CoMo synthesized catalysts. (a) CoMo/γ-Al_2O_3, (b) CoMo-AA/γ-Al_2O_3, (c) CoMo-EDTA/γ-Al_2O_3, (d) CoMo-CA/γ-Al_2O_3.

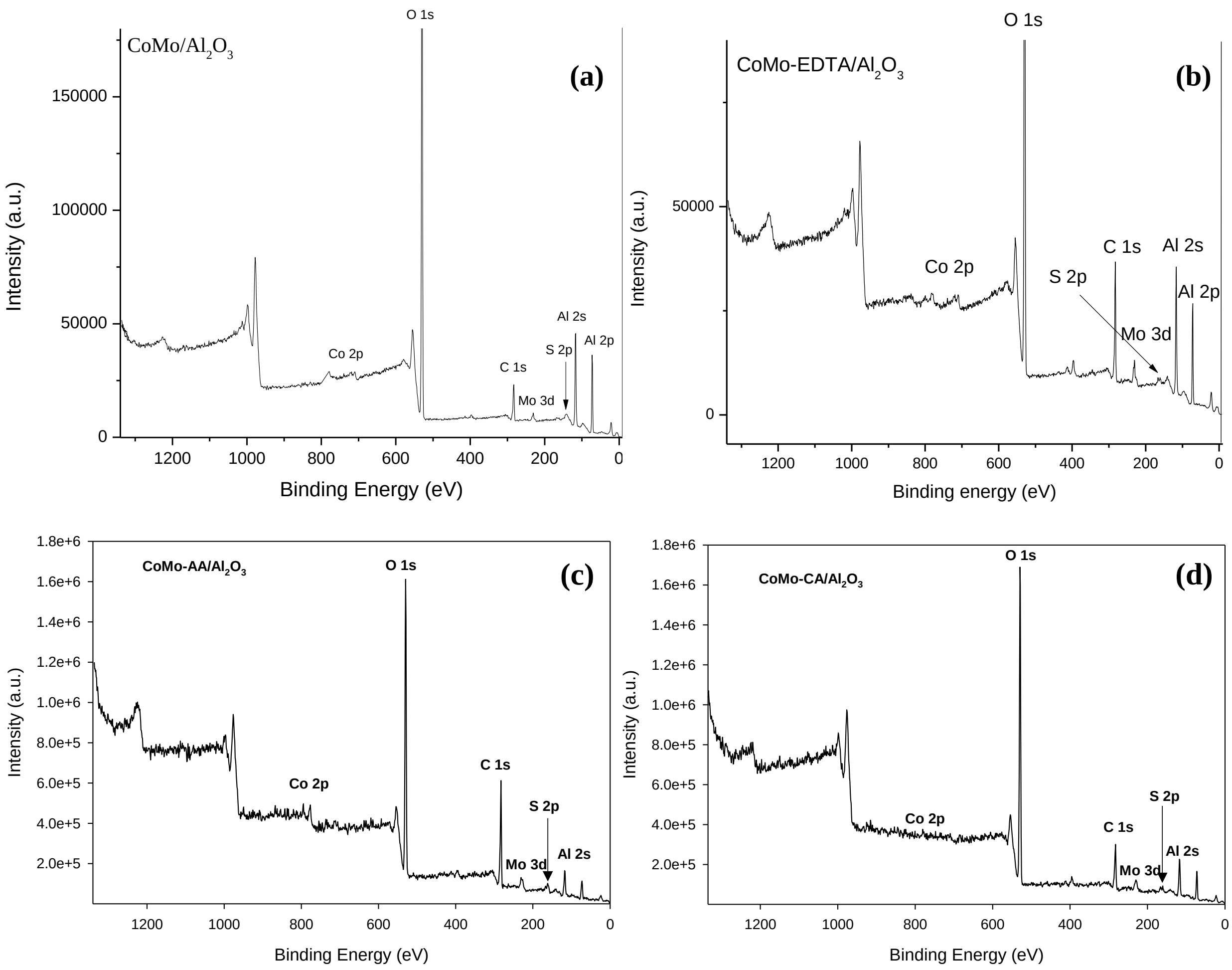

Fig. S7. XPS spectra for (a) CoMo/γ-Al_2O_3, (b) CoMo-EDTA/γ-Al_2O_3, (c) CoMo-AA/γ-Al_2O_3, (d) CoMo-CA/γ-Al_2O_3 survey spectrum.

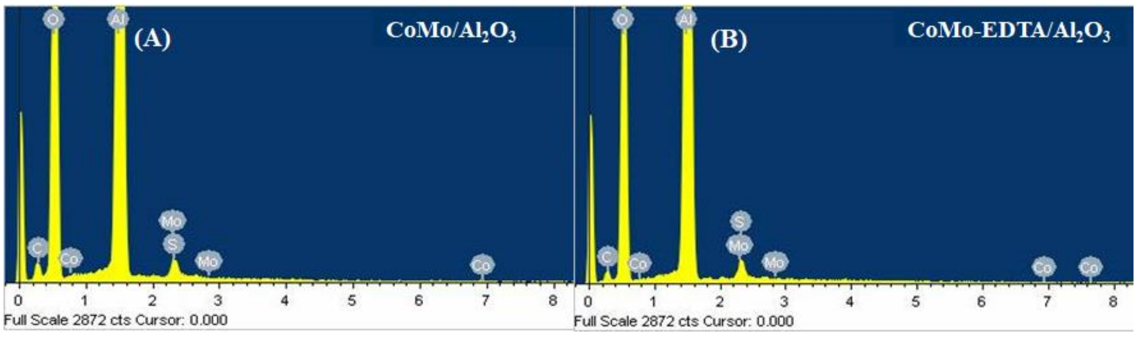

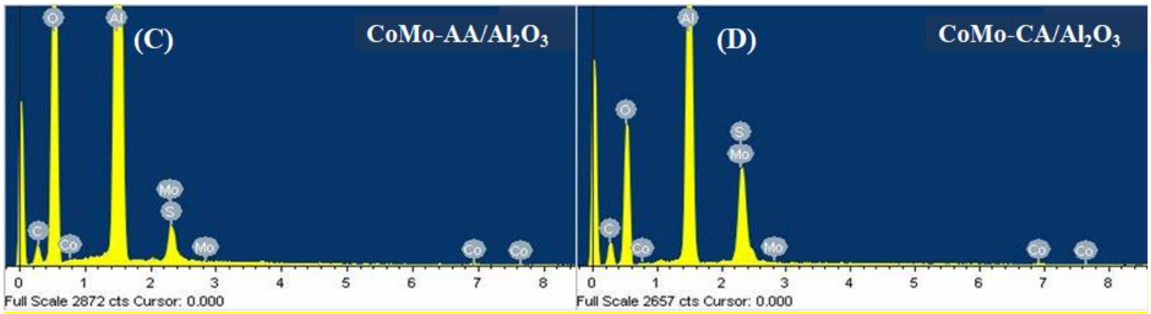

Fig. S8. EDS analysis for (A) CoMo/γ-Al_2O_3, (B) CoMo-EDTA/γ-Al_2O_3, (C) CoMo-AA/γ-Al_2O_3, (D) CoMo-CA/γ-Al_2O_3 catalysts.

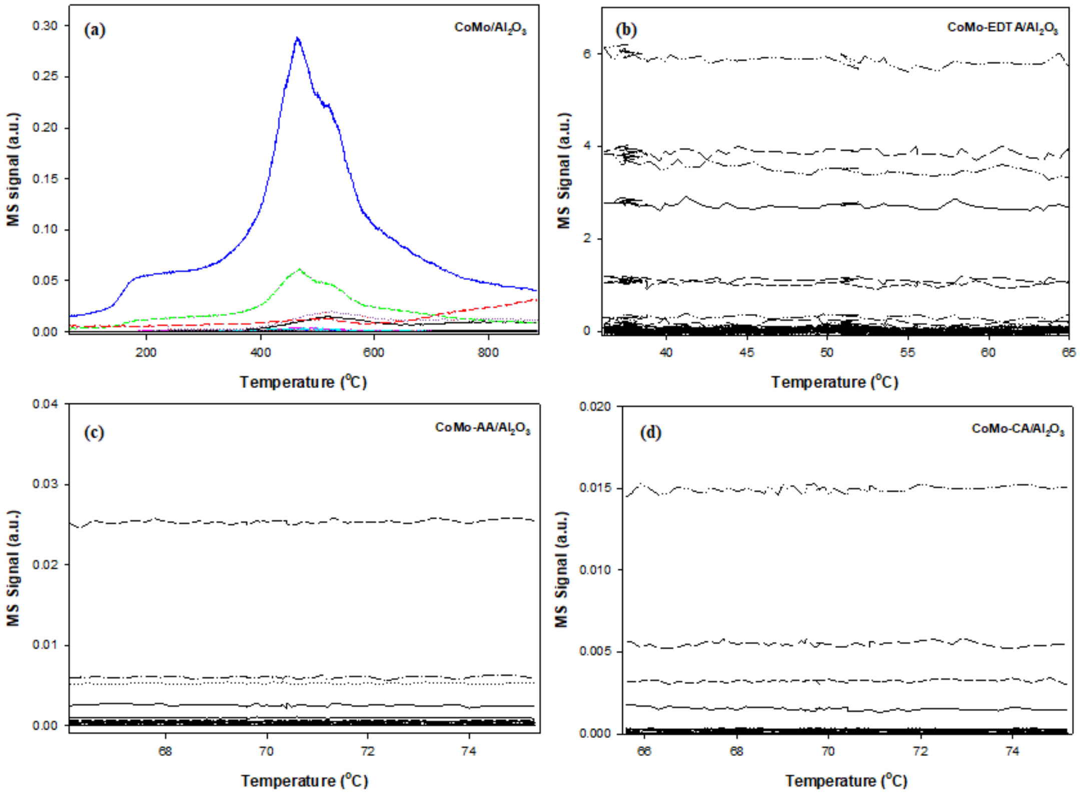

Figure S9: MS signals for sulfided (a) CoMo/γ-Al_2O_3, (b) CoMo-EDTA/γ-Al_2O_3, (c) CoMo-AA/γ-Al_2O_3, and (d) CoMo-CA/γ-Al_2O_3.

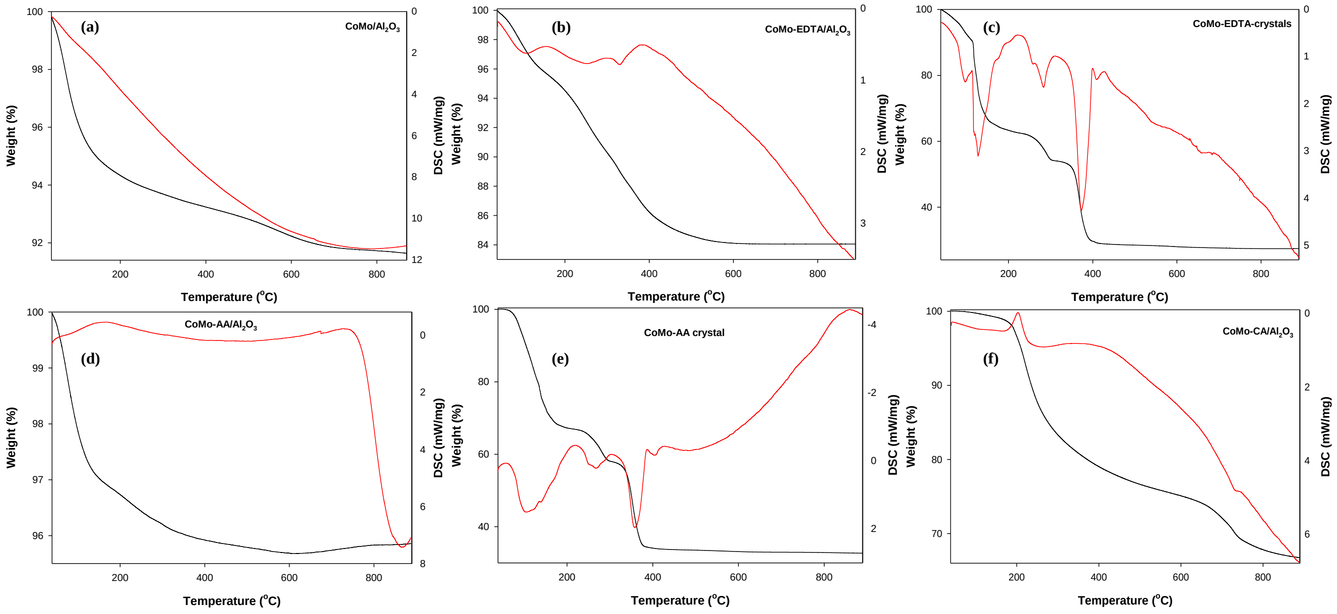

Figure S10: TGA-DSC for (a) CoMo/γ-Al_2O_3, (b) CoMo-EDTA/γ-Al_2O_3, (c) CoMo-EDTA-crystal, (d) CoMo-AA/γ-Al_2O_3, (d) CoMo-AA-crystal, and (f) CoMo-CA/γ-Al_2O_3.

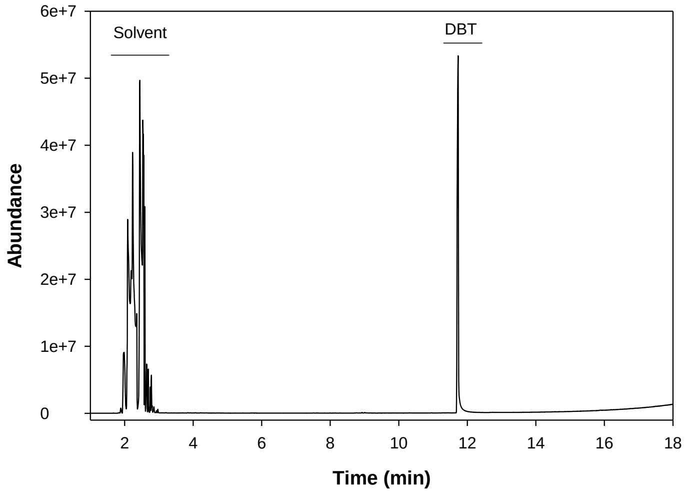

Figure S11: GC chromatogram of DBT before HDS.

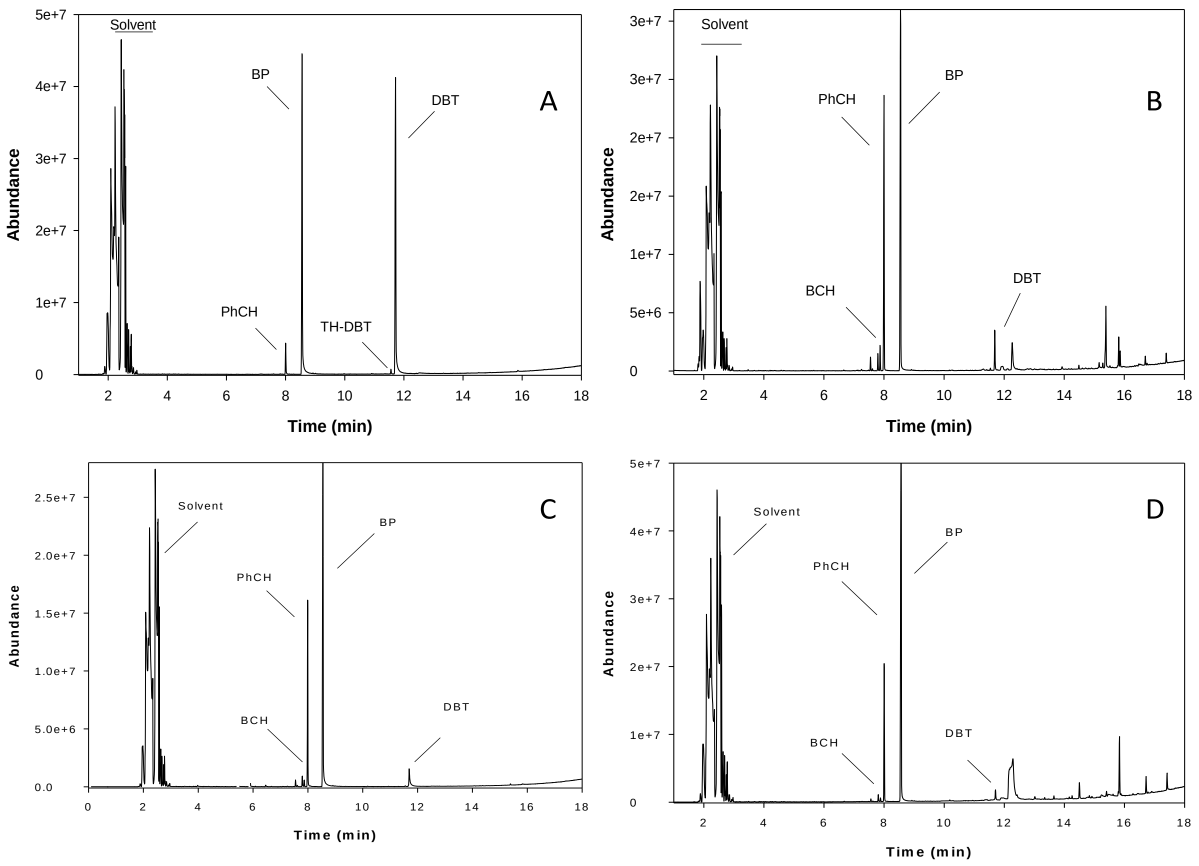

A
Solvent
BP
DBT
PhCH
TH-DBT
B
PhCH
BCH
C
D
Abundance
Time (min)

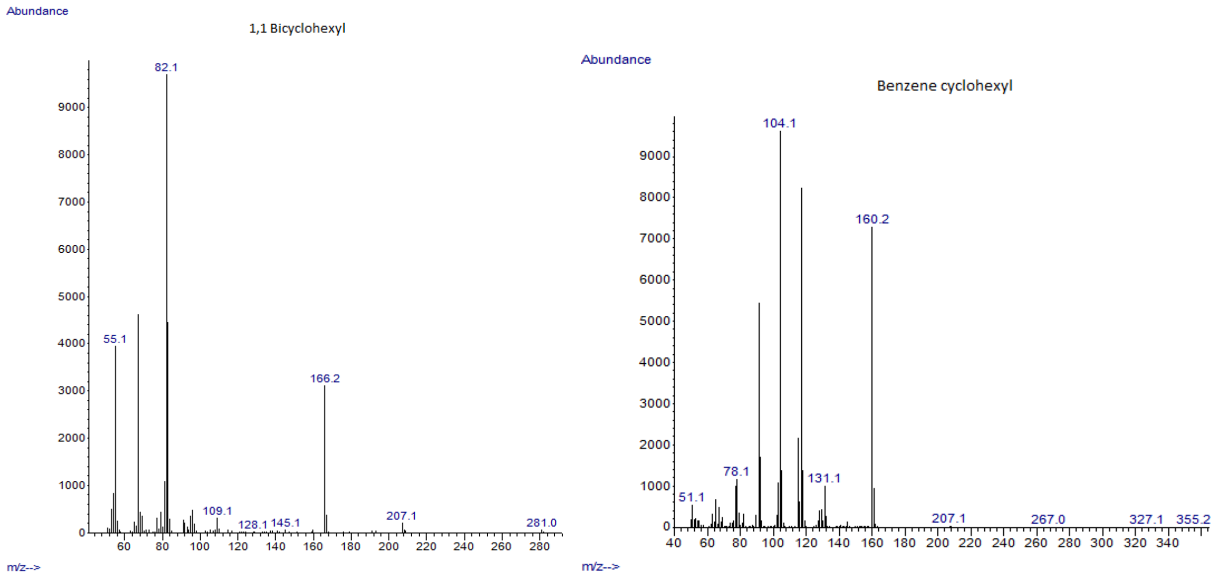

Abundance
1,1 Bicyclohexyl
82.1
55.1
109.1
128.1
145.1
166.2
207.1
281.0
m/z-->
Benzene cyclohexyl
104.1
160.2
51.1
78.1
131.1
267.0
327.1
355.2

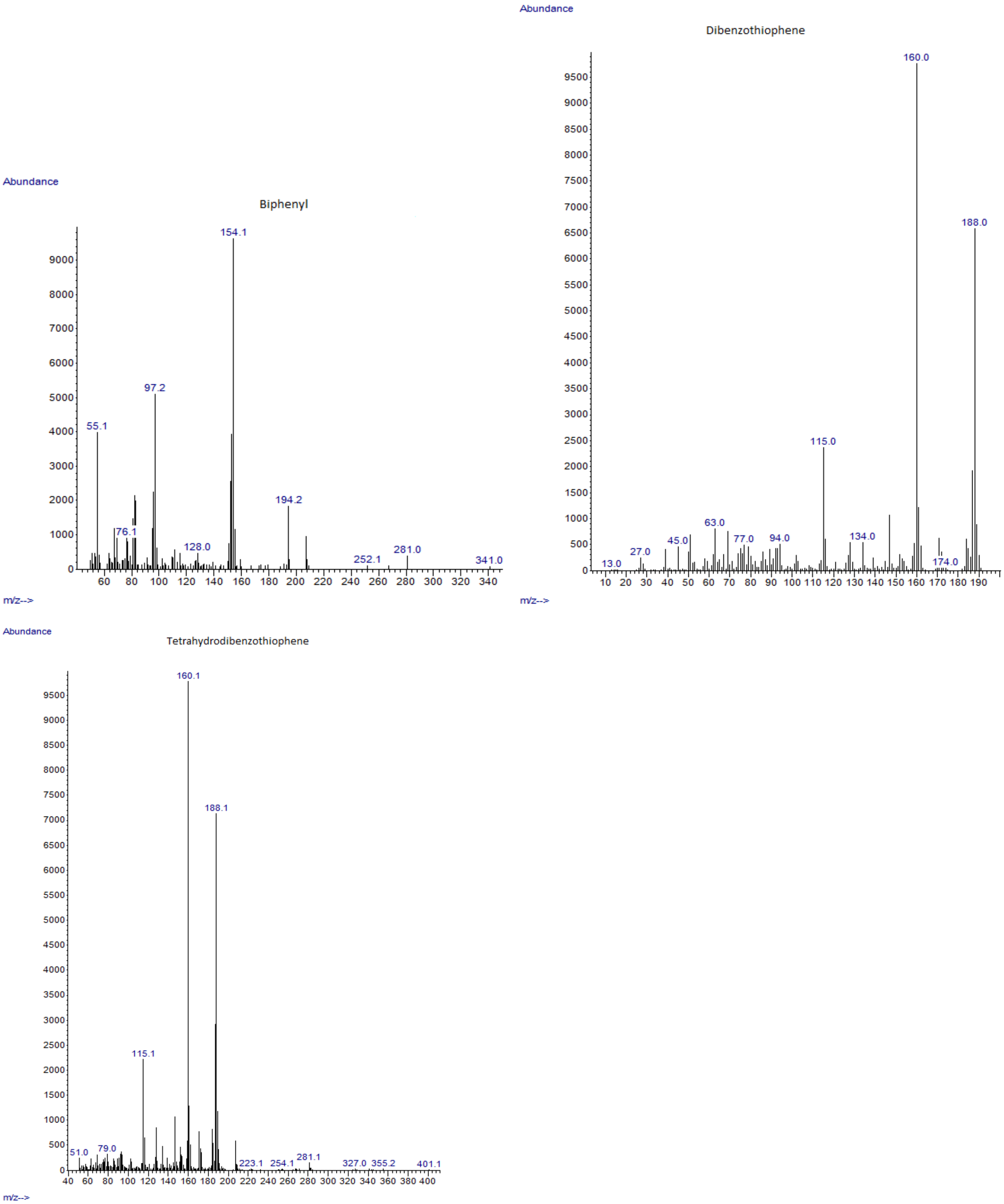

Fig. S12. GC chromatogram of DBT after HDS using (A) CoMo/γ-Al_2O_3, (B) CoMo-EDTA/γ-Al_2O_3, (C) CoMo-AA/γ-Al_2O_3, and (D) CoMo-CA/γ-Al_2O_3. GCMS data for BP = biphenyl, PhCH = biphenyl cyclohexane, BCH = bicyclohexyl, TH-DBT = tetrahydrodibenzothiophene is also provided.

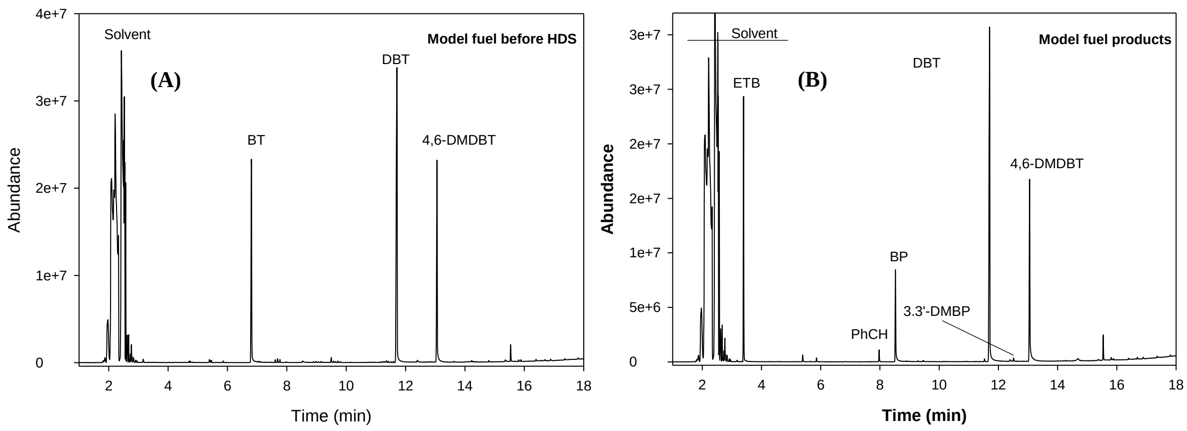

Figure S13: GC chromatogram for BT, DBT, 4,6-DMDBT (a) Model fuel before HDS and (b) Model fuel after HDS activity using CoMo-CA/Al_2O_3 catalyst. BT = benzothiophene, DBT = dibenzothiophene, 4,6-DMDBT = 4,6-dimethyldibenzothiophene, 3.3'DMBP = 3.3'-dimethylbiphenyl, BP = biphenyl, PhCH = biphenyl cyclohexane, ETD = ethlybenzene.

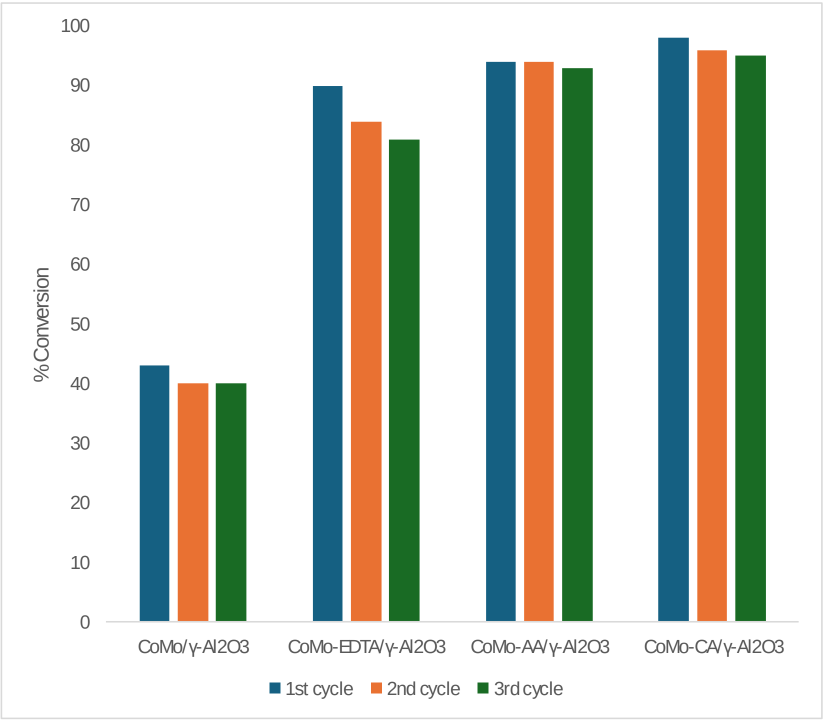

Figure S14. The HDS performance of DBT on CoMo-CA/γ-Al_2O_3, CoMo-EDTA/γ-Al_2O_3, CoMo/γ-Al_2O_3 and CoMo-AA/γ-Al_2O_3 in 3 cycles.

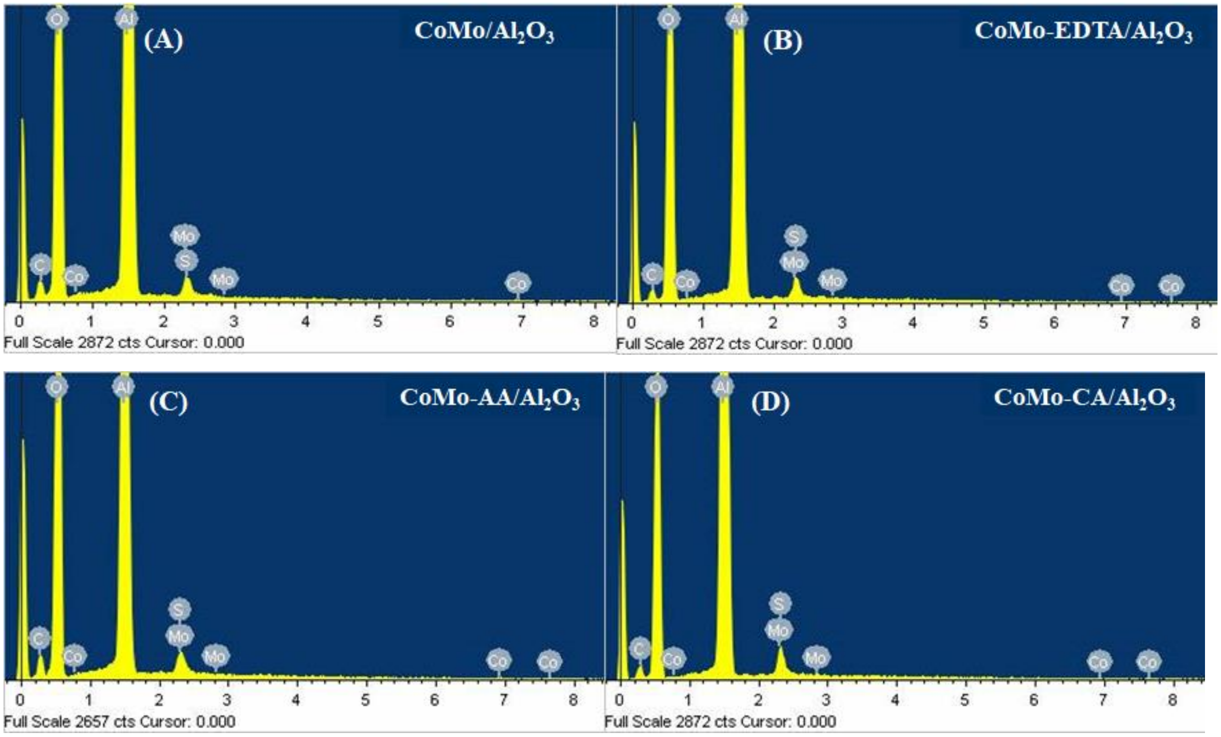

Figure S15 EDS analysis of (a) CoMo/Al_2O_3, (b) CoMo-EDTA/Al_2O_3, (c) CoMo-AA/Al_2O_3, (d) CoMo-CA/Al_2O_3 catalysts after three HDS cycles.

Table S1: Selected bond distances (Å) in the crystal structure of CoMo-EDTA.

Atoms	Distance	Atoms	Distance
**Mo(1)–O(8)**	1.712(3)	Co(1)–N(2)	2.275(5)
**Mo(1)–O(9)**	1.934(2)	Co(1)–O(1[a])	2.142(4)
**Mo(1)–O(10)**	2.249(3)	Co(1)–O(3[a])	2.106(5)
**Mo(1)–O(11)**	2.157(3)	Na(1)–O(1)	2.487(4)
**Mo(1)–O(23)**	1.712(3)	Na(1)–O(5)	2.346(5)
**Mo(1)–O(27)**	1.981(3)	Na(1)–O(6)	2.345(4)
**Mo(2)–O(11)**	2.171(2)	Na(1)–O(7)	2.334(5)
**Mo(2)–O(12)**	1.709(3)	Na(1)–O(1[a])	2.487(4)
**Mo(2)–O(13)**	1.728(3)	Na(1)–O(6[a])	2.345(4)
**Mo(2)–O(14)**	1.920(3)	O(1)–C(1)	1.233(6)
**Mo(2)–O(27)**	1.914(3)	O(2)–C(1)	1.238(7)
**Mo(2)–O(19[b])**	2.502(2)	O(3)–C(7)	1.224(8)
**Mo(3)–O(11)**	2.148(3)	O(4)–C(7)	1.237(9)
**Mo(3)–O(14)**	1.978(3)	N(1)–C(2)	1.453(6)
**Mo(3)–O(15)**	2.275(3)	N(1)–C(3)	1.497(9)
**Mo(3)–O(16)**	1.707(4)	N(1)–C(2[a])	1.453(6)
**Mo(3)–O(17)**	1.713(3)	N(1)–C(3[a])	1.497(9)
**Mo(3)–O(18)**	1.931(2)	N(2)–C(4)	1.466(9)
**Mo(4)–O(10)**	1.906(3)	N(2)–C(5)	1.459(12)
**Mo(4)–O(11)**	2.254(2)	N(2)–C(6)	1.448(13)
**Mo(4)–O(15)**	1.885(3)	N(2)–C(4[a])	1.466(9)
**Mo(4)–O(19)**	1.739(2)	N(2)–C(5[a])	1.459(12)
**Mo(4)–O(11[b])**	2.254(2)	N(2)–C(6[a])	1.448(13)
**Mo(4)–O(19[b])**	1.739(2)	O(5)–H(5)	0.8400
**Co(1)–O(1)**	2.142(4)	O(5)–H(5[a])	0.8400
**Co(1–O(3)**	2.106(5)	O(6)–H(6B)	0.8000
**Co(1)–O(5)**	2.277(4)	O(6)–H(6A)	0.8800
**Co(1)–N(1)**	2.280(5)	O(7)–H(7A)	0.9600

Table S2: Selected bond angles (°) in crystal structure of CoMo-EDTA

Atoms	Angle	Atoms	Angle
**O(3[a])–Co(1)–O(5)**	79.54(13)	O(14)–Mo(2)–O(19[b])	80.29(12)
**N(1)–Co(1)–N(2)**	77.15(17)	O(19b)–Mo(2)–O(27)	79.92(12)
**O(1[a])–Co(1)–N(1)**	76.18(13)	O(11)–Mo(3)–O(14)	73.03(11)
**O(3a)– Co(1)–N(1)**	116.70(13)	O(11)–Mo(3)–O(15)	71.82(10)
**O(1[a])–Co(1)–N(2)**	130.30(11)	O(11)–Mo(3)–O(16)	94.26(12)
**O(3a)–Co(1)–N(2)**	74.50(15)	O(11)–Mo(3)–O(17)	157.58(14)
**O(1 [a])–Co(1)–O(3[a])**	155.15(15)	O(11)–Mo(3)–O(18)	87.74(15)
**O(1)–Co1–O(3[i])**	81.26(19)	O(14)–Mo(3)–O(15)	84.08(12)
**O(3)–Co(1)–O(5)**	79.54(13)	O(14)–Mo(3)–O(16)	100.76(15)
**O(3)–Co(1)–N(1)**	116.70(13)	O(14)–Mo(3)–O(17)	92.09(15)
**O(3)–Co(1)–N(2)**	74.50(15)	O(14)–Mo(3)–O(18)	154.17(15)
**O(1[a])–Co(1)–O(3)**	81.26(19)	O(15)–Mo(3)–O(16)	163.42(14)
**O(3)–Co(1)–O(3[a])**	108.2(2)	O(15)–Mo(3)–O(17)	90.39(15)
**O(5)–Co(1)–N(1)**	148.02(14)	O(15)–Mo(3)–O(18)	73.42(12)
**O(5) –Co(1) –N(2)**	134.83(17)	O(16)–Mo(3)–O(17)	105.15(16)
**O(1) –Co(1) –O(5)**	79.77(12)	O(16)–Mo(3)–O(18)	97.66(15)
**O(1) –Co(1) –N(1)**	76.18(13)	O(17)–Mo(3)–O(18)	100.43(18)
**O(1) –Co(1) –O(3)**	155.15(15)	O(10)–Mo(4)–O(11)	76.82(11)
**O(1) –Co(1) –O(5)**	79.77(12)	O(10)–Mo(4)–O(15)	143.39(16)
**O(1) –Co(1) –N(2)**	130.30(11)	O(10)–Mo(4)–O(19)	100.66(12)
**O(1) –Co(1) –O(1[a])**	81.66(15)	O(10)–Mo(4)–O(11[b])	76.82(11)
**O(8) –Mo(1) –O(9)**	97.45(14)	O(10)–Mo(4)–O(19[b])	100.66(12)
**O(8) –Mo(1) –O(10)**	162.98(14)	O(11)–Mo(4)–O(15)	77.15(12)
**O(8) –Mo(1) –O(11)**	93.25(12)	O(11)–M(4)–O(19)	171.77(9)

Table S2: Continued

Atoms	Angle	Atoms	Angle
**O(8) –Mo(1) –O(23)**	104.00(16)	O(11)–Mo(4)–O(11^{b})	88.36(7)
**O(8) –Mo(1) –O(27)**	99.82(14)	O(11)–Mo(4)–O(19^{b})	83.43(9)
**O(9) –Mo(1) –O(10)**	73.80(12)	O(15)–Mo(4)–O(19)	101.44(13)
**O(9) –Mo(1) –O(11)**	88.33(15)	O(11^{b})–Mo(4)–O(15)	77.15(12)
**O(9) –Mo(1) –O(23)**	100.15(18)	O(15)–Mo(4)–O(19^{b})	101.44(13)
**O(9) –Mo(1) –O(27)**	155.26(15)	O(11^{b})–Mo(4)–O(19)	83.43(9)
**O(10) –Mo(1) –O(11)**	72.26(10)	O(19)–Mo(4)–O(19^{b})	104.78(10)
**O(10) –Mo(1) –O(23)**	92.06(15)	O(11^{b})–Mo(4)–O(19^{b})	171.77(9)
**O(10) –Mo(1) –O(27)**	84.78(12)	O(12) –Mo(2) –O(19^{a})	79.51(11)
**O(11) –Mo(1) –O(23)**	159.49(14)	O(15) –Mo(4) –O(19)	101.44(13)
**O(11) –Mo(1) –O(27)**	73.18(10)	O(11^{b}) –Mo(4) –O(15)	77.15(12)
**O(23) –Mo(1) –O(27)**	92.72(15)	O(15) –Mo(4) –O(19^{b})	101.44(13)
**O(11) –Mo(2) –O(12)**	149.22(11)	O(11^{b}) –Mo(4) –O(19)	83.43(9)
**O(11) –Mo(2) –O(13)**	105.83(11)	O(19) –Mo(4) –O(19^{b})	104.78(10)
**O(11) –Mo(2) –O(14)**	73.59(13)	O(11^{b}) –Mo(4) –O(19^{b})	171.77(9)
**O(11) –Mo(2) –O(27)**	74.13(13)		
**O(11)–Mo(2) –O(19^{b})**	69.71(7)		
**O(12) –Mo(2) –O(13)**	104.95(13)		
**O(12) –Mo(2) –O(14)**	101.33(18)		
**O(12) –Mo(2) –O(27)**	101.47(18)		
**O(13) –Mo(2) –O(14)**	98.76(18)		
**O(13) –Mo(2) –O(27)**	98.87(18)		
**O(13)–Mo(2) –O(19^{b})**	175.54(11)		
**O(14) –Mo(2) –O(27)**	146.36(14)		

Table S3: Hydrogen bond geometry for CoMo-EDTA in crystal packing.

**Atoms**	**D-H... Å**	**H…A/Å**	**D…A/Å**	**D-H... A/°**	**Symmetry code**
O(7)-H(7A)-O(21)	0.9600	2.0800	2.988 (9)	161.00	-
O(20)-H(20A)-O(19)	0.9700	1.9600	2.897 (5)	163.00	1-x, y, z
O(22)-H(22A)-O(27)	0.9500	1.8500	2.798 (6)	173.00	-
O(22)-H(22B)-O(2)	1.0100	1.8600	2.871 (6)	177.00	-x, -1+y, z
O(24)-H(24A)-O(2)	1.0400	1.7300	2.764 (6)	174.00	-
O(24)-H(24B)-O(8)	1.0300	1.9800	2.939 (5)	153.00	**-**
O(25)-H(25A)-O(1)	0.9600	2.0300	2.902 (6)	149.00	½-x,2-y, ½ +z
O(25)-H(25B)-O(8)	0.8200	2.3900	2.877 (7)	119.00	-
O(28)-H(28B)-O(13)	0.9400	1.9200	2.783 (5)	152.00	-
O(29)-H(29A)-O(30)	1.0000	2.3200	3.189 (11)	144.00	-

Table S4: Selected bond distances (Å).

Atoms	Distance	Atoms	Distance
**Co(1)–O(1)**	2.0962(8)	O(3)–H(3A)	0.829(10)
**Co(1)–O(3)**	2.0892(10)	O(3)–H(3B)	0.831(10)
**Co(1)–O(4)**	2.1195(9)	O(4)–H(4A)	0.840(17)
**Co(1)–O(1^{i})**	2.0962(8)	O(4)–H(4B)	0.833(15)
**Co(1)–O(3^{i})**	2.0892(10)	C(1)–C(2)	1.4987(19)
**Co(1)–O(4^{i})**	2.1195(9)	C(2)–H(2A)	0.9600
**O(1)–C(1)**	1.2732(15)	C(2)–H(2B)	0.9600
**O(2)–C(1)**	1.2488(15)	C(2)–H(2C)	0.9600

Table S5: Selected bond angles (°).

Atoms	Angle	Atoms	Angle
**O(1)–Co(1)–O(3)**	90.57(4)	O(3)–Co(1)–O(3^i)	180.00
**O(1)–Co(1)–O(4)**	90.18(3)	O(3)–Co(1)–O(4^i)	90.66(4)
**O(1)–Co(1)–O(1^i)**	180.00	O(1^i)–Co(1)–O(4)	89.82(3)
**O(1)–Co(1)–O(3^i)**	89.43(4)	O(3^i)–Co(1)–O(4)	90.66(4)
**O(1)–Co(1)–O(4^i)**	89.82(3)	O(4)–Co(1)–O(4^i)	180.00
**O(3)–Co(1)–O(4)**	89.34(4)	O(1^i)–Co(1)–O(3^i)	90.57(4)
**O(1^i)–Co(1)–O(3)**	89.43(4)	O(1^i)–Co(1)–O(4^i)	90.18(3)
**O(3^i)–Co1–O(4^i)**	89.34(4)		

Table S6: Hydrogen bond geometry for complex Co-AA crystal.

**Atoms**	**D-H…..Å**	**H….A/Å**	**D….A/Å**	**D-H…A/°**	**Symmetry code**
O(3)-H(3A)-O(4)	0.829(10)	2.086(13)	2.8378(13)	150.7(15)	1+x,y,z
O(3)-H(3B)-O(2)	0.831(10)	1.845(13)	2.6360(14)	159(2)	-
O(4)-H(4A)-O(2)	0.840(17)	1.847(16)	2.7103(13)	173.7(18)	1-x,-1/2+y,3/2-z
O(4)-H(4B)-O(1)	0.833(15)	1.925(15)	2.7243(12)	160.6(15)	1+x,y,z
C(2)-H(2A)-O(3)	0.9600	2.5500	3.4672(19)	160.00	1-x,1/2+y,3/2-z

Table S7. EDS Qualitative atomic percentage of Co, C, O, S and Mo for sulfided HDS catalysts.

**Catalysts**	**Atomic Percentage (wt. %)**						**S/Mo**	**C/Mo**
	C K	O K	Al K	S K	Co K	Mo L		
**CoMo/γ-Al_2O_3**	13.50	60.21	25.03	0.79	0.11	0.36	2.19	37.50
**CoMo-EDTA/γ-Al_2O_3**	12.34	56.82	29.68	0.69	0.13	0.33	2.09	37.39
**CoMo-AA/γ-Al_2O_3**	32.32	46.52	18.47	3.28	0.15	1.27	2.58	25.45
**CoMo-CA/γ-Al_2O_3**	10.27	61.13	26.80	0.70	0.10	0.34	2.06	30.21

Table S8. EDS Qualitative atomic percentage of Co, C, O, S and Mo after three HDS cycles.

	Atomic Percentage (wt. %)							
	C K	O K	Al K	S K	Co K	Mo L	S/Mo	C/Mo
**CoMo/γ-Al_2O_3**	9.60	60.73	28.91	0.51	0.07	0.18	2.83	53.3
**CoMo-EDTA/ γ-Al_2O_3**	14.06	60.02	25.33	0.25	0.06	0.29	0.86	48.5
**CoMo-AA/ γ-Al_2O_3**	17.00	60.18	19.91	1.94	0.13	1.26	1.53	13.4
**CoMo-CA/γ-Al_2O_3**	8.16	62.16	28.70	0.67	0.07	0.25	2.68	32.6
